# Mixed-effect Bayesian network reveals personal effects of nutrition

**DOI:** 10.1038/s41598-021-91437-3

**Published:** 2021-06-08

**Authors:** Jari Turkia, Lauri Mehtätalo, Ursula Schwab, Ville Hautamäki

**Affiliations:** 1 School of Computing, University of Eastern Finland, 80101 Joensuu, Finland; 2 CGI Suomi Oy, Joensuu, Finland; 3 Natural Resources Institute Finland (Luke), Bioeconomy and Environment Unit, Yliopistokatu 6, 80101 Joensuu, Finland; 4 School of Medicine, Institute of Public Health and Clinical Nutrition, University of Eastern Finland, Kuopio, Finland; 5 Department of Medicine, Endocrinology and Clinical Nutrition, Kuopio University Hospital, Kuopio, Finland

**Keywords:** Nutrition, Bayesian inference

## Abstract

Nutrition experts know by their experience that people can react very differently to the same nutrition. If we could systematically quantify these differences, it would enable more personal dietary understanding and guidance. This work proposes a mixed-effect Bayesian network as a method for modeling the multivariate system of nutrition effects. Estimation of this network reveals a system of both population-wide and personal correlations between nutrients and their biological responses. Fully Bayesian estimation in the method allows managing the uncertainty in parameters and incorporating the existing nutritional knowledge into the model. The method is evaluated by modeling data from a dietary intervention study, called Sysdimet, which contains personal observations from food records and the corresponding fasting concentrations of blood cholesterol, glucose, and insulin. The model’s usefulness in nutritional guidance is evaluated by predicting personally if a given diet increases or decreases future levels of concentrations. The proposed method is shown to be comparable with the well-performing Extreme Gradient Boosting (XGBoost) decision tree method in classifying the directions of concentration increases and decreases. In addition to classification, we can also predict the precise concentration level and use the biologically interpretable model parameters to understand what personal effects contribute to the concentration. We found considerable personal differences in the contributing nutrients, and while these nutritional effects are previously known at a population level, recognizing their personal differences would result in more accurate estimates and more effective nutritional guidance.

## Introduction

Nutrition is an important contributor to good health, and dietary factors affect the risk of common diseases, such as type 2 diabetes^1^, cardiovascular diseases^1^, and cancer^2^. Dietary recommendations are based on the general effects of nutrients at the population level. However, there is an increasing body of knowledge that the effects of nutrients may vary largely between individuals^3^. This observation has led to an increased interest in personalized nutrition where information on personal characteristics is used to develop targeted nutritional guidance^4,5^.

Personally targeted nutritional guidance has shown to be more effective than guidance at a general level based on common recommendations. Food4Me study^4^ provided strong evidence for the effect of personal guidance. Increased knowledge of inter-individual variations optimized the results of nutritional guidance in Food4Me study, and it showed also that the increased personal information motivates also patients themselves. In a more specific case, Zeevi et al.^6^ could predict personal glucose response and use this information in selecting personal diets. Clear statistical indicators of personal reactions would be a significant aid in targeted nutritional guidance.

The computational approaches for diet recommendations in both Food4Me^4^ and the glucose metabolism prediction^6^ were based on decision trees. In Food4Me, the decision trees were constructed manually based on expert knowledge, while the decision trees for optimizing the diet for glucose metabolism were constructed algorithmically with boosted decision trees from observational data. Decision trees may perform well in classification, but we argue that a statistical model that provides biologically interpretable parameters and manages uncertainty in parameter estimations would be more useful in understanding and drawing conclusions on the complex effects of nutrition.

Bayesian networks have been found to be useful in learning the structures of large interconnected systems like gene interactions^7^, lifestyle predictors of obesity^8^ and causalities of attention-deficit/hyperactivity disorder (ADHD)^9^. Bayesian network is a directed graph of interconnected random variables, and unlike decision trees, it allows conditioning on any of these variables once the graph is estimated^10^. In the context of nutritional guidance this belief propagation^11^ could be used in conditional reasoning of nutrient intake levels once the evidence from personal reactions is gathered.

We propose a mixed-effect Bayesian network as an appealing method for modeling the connections between nutrients and their biological responses. Mixed-effect parameterization^12,13^ allows studying the effects of nutrition in both general and personal levels, and implementing it as a Bayesian hierarchical model^14^ allows managing the uncertainty in all parameter estimates. Our procedure for creating the Bayesian network was to give a biologically motivated preliminary graph structure and then learn the meaningful effects and their magnitudes from observational data. We did not want to restrict the model too tight on previously known effects but also leave a possibility to find new connections between the nutrients and the blood concentrations. The structure of our graph is inspired by the systems biology of personal nutrition described by Boorsma et al.^3^. They illustrate a system that starts from nutrients at a person’s diet and results in personally different biological responses. In this system, the nutrients participate in different biological processes, continue to affect several organs, and finally manifest themselves in responses like different blood characteristics. This layered behavior can be naturally modeled with a Bayesian network where nutrients and their biological responses are represented as connected random variables. In our model, we also enforced this assumption that the nutrients have directed connections towards the concentrations, but not the other way around. For now, we observe only the nutrient intake and the personal responses in resulting concentrations, but the network could be expanded to model the biological processes causing these responses. The model parameters are intended to have a clear biological interpretation that helps confirming the findings against the existing clinical knowledge. The nutritional effects are presented in an additive scale with clearly separated general and personal effects.

The aim of this work is to model the personal responses on blood concentrations and indicate the nutrients that contribute to these concentrations. The model is aimed for personal nutritional guidance and its usefulness in this context is evaluated by predicting personally if a given dietary modifications will increase or decrease the responses in concentrations. This classification test allows comparing our method with the existing decision tree methods that can only be used in classifying the diet options. In addition to classification, our model also reveals the full probability distributions for nutrient levels that personally contribute to the concentrations. This can be used in reasoning the personally adjusted nutrient intakes given their estimated effects. By applying our method to nutritional data, we have succeeded in finding the expected personal differences in reactions and they are shown to be even opposite between persons. Furthermore, these reactions are shown to form clusters of similarly reacting persons.

## Description of personal nutrition data

For evaluating our method, we have used personal nutrition data from a dietary intervention study called Sysdimet^15^. This randomized controlled trial data included food records, personal information, and laboratory results from 106 subjects with impaired glucose metabolism and features of the metabolic syndrome. The data were gathered in four repeated time points during 12 weeks. At the beginning of the trial, the subjects kept a 4-day food record, and personal concentrations were measured. During the weeks 3, 7, and 11 of the trial, the subjects kept food records and new blood samples were drawn within a week after that at weeks 4, 7, and 12. This gave a 1 week response time for the food record observation to show in the first blood measurement.

**Figure 1:**
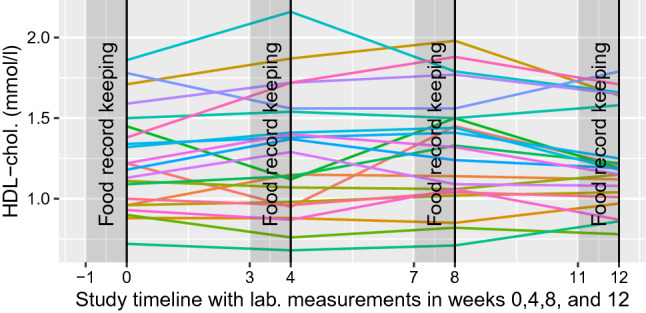
The progress of HDL cholesterol concentration for 10 out of all 106 subjects during the 12-week study in the Sysdimet data. The concentrations are measured at weeks 0, 4, 8, and 12 and the food records are kept in a week before the measurement. The figure is plotted with ggplot2 package for R language (v 3.3.2, https://ggplot2.tidyverse.org).

For predicting the personal reactions we selected sex, a cholesterol-lowering medication, and 20 different nutrients as possible predictors of change at the blood concentrations. Goals of the personal nutrition may vary, but in this example, we considered the personal blood concentrations of total serum cholesterol, HDL-cholesterol, LDL-cholesterol, insulin, and plasma glucose. The goal would be then reaching the reference values of these concentrations. The considered nutrients were selected based on the existing clinical knowledge for possibly reacting with the concentrations. Preliminary exploration of the HDL-concentration data in Fig. 1 shows that there are personal differences both in levels and in changes of concentrations during the observed 12-week period. Similar trends can be seen also in other measured concentrations. Plots of all considered concentrations are provided in Supplementary Fig. S1.

These data provide an initial setting for the Bayesian network with five responses and 22 possible predictors for each of them, making 110 different possible effects between nutrients and concentrations. This network corresponds to the systems biological framework^3^ where nutrients affect bodily responses. Only now, we don’t have information about the bodily processes and organs responsible for the effect. A list of the considered nutrients and the network setting is seen in Fig. 3.

In the original Sysdimet trial, the subjects were randomly assigned into groups for studying the effects of different diets^15^. Subjects at the different groups were still assumed to react similarly to the same nutrition and the original grouping was omitted in this analysis. Possible personal differences in the reactions were unknown, and those are our goal to estimate.

## Mixed-effect Bayesian network for effects of nutrition

A thorough overview of Bayesian networks, and probabilistic graphical models in general, can be found at the works of Pearl^10^, Koller and Friedman^16^, Bishop^17^, and Nagarajan and Scutari^18^. Overview of classical univariate and multivariate mixed-effects models can be found e.g. in Mehtätalo and Lappi^13^. Blei^19^ describes an iterative procedure of graphical model development and criticism that is followed here. First, a simple model is formulated based on the personal variations that are believed to exist in data. Then, given the data, an approximate posterior is inferenced for the model candidate and it is tested against the data to see if the model is useful or biased. Finally, the model is revised until criteria are met and improvement can be done.

### Model specification

Let us denote data with food records and concentration measurements with *D* and a Bayesian network modeling the data with *G*. The posterior probability of the network *G* can be decomposed into a product of data likelihood given the network and a prior probability of that network

1 $$\begin{aligned} P(G|D) \propto P(D|G)P(G). \end{aligned}$$

The conditional likelihood of data factorizes further into *v* independent local distributions of blood concentrations according to their Markov blankets and data are assumed to be grouped by $k = 1,\dots ,s$ subjects with $n_k$ observations for each subject

2 $$\begin{aligned} \begin{aligned} P(D|G)&= \prod _{i=1}^v P(D|G_i) \\&= \prod _{d=1}^{n_k} \prod _{k=1}^s \prod _{i=1}^v P(Y_{ikd} | X_{jkd} = pa_j(Y_{ik})_d, G_i, \mu _{ikd}, \varvec{\phi }_i), j = 1,\dots ,p, \end{aligned} \end{aligned}$$

where $Y_i$ is a random variable for an *i*th concentration response, and $pa(Y_i)$ denotes the set of *p* observed parent variables for $Y_i$ in the directed graph $G_i$. Parent variables are direct predecessors of variables in a directed graphical model. Furthermore, $\mu _{ikd}$ is an expected value of the concentration for a personal observation and set $\varvec{\phi }_i$ pools general parameters of the distribution.

The expected value of the concentration $Y_{ik}$ for the subject *k* is then defined then with a linear predictor that is divided into general and personal parts

3 $$\begin{aligned} \begin{aligned} \varvec{\mu }_{ik}&= \varvec{X}_{ik}\varvec{\beta }_i + \varvec{Z}_{ik}\varvec{b}_{ik}, \\ \varvec{X}_{ik}&= pa_X(Y_{ik}), \varvec{Z}_{ik} = pa_Z(Y_{ik}) \\ {\varvec{b}_i}_k&\sim \mathcal {N}(0, \varvec{\Sigma }_{b_{ik}}), \\ \varvec{\Sigma }_{b_{ik}}&= \varvec{T}_{ik} \varvec{C}_{ik} \varvec{T}_{ik}' = \varvec{T}_{ik} \varvec{L}_{ik} \varvec{L}_{ik}' \varvec{T}_{ik}', \\ \varvec{T}_{ik}&= diag({\sigma _{b_i}^{(1)}},\dots ,{\sigma _{b_i}^{(p)}}), \end{aligned} \end{aligned}$$

where operator $pa_X()$ picks the values of observed parent variables from the food record data as a model matrix, and $pa_Z()$ picks a similar model matrix for subject *k* of those food record variables that are assumed to have personal differences between subjects. The model’s assumptions of personal differences are elaborated in the following sections. Furthermore, the variance-covariance matrix ${\varvec{\Sigma }_b}_{ik}$ of personal effects $\varvec{b}_{ik}$ is defined with a diagonal matrix $\varvec{T}_{ik}$ containing personal effect standard deviations and personal effect correlation matrix $\varvec{C}_{ik}$ that decomposes into triangular Cholesky matrix $\varvec{L}_{ik}$. These matrices are unique to each response variable $Y_i$.

**Figure 2:**
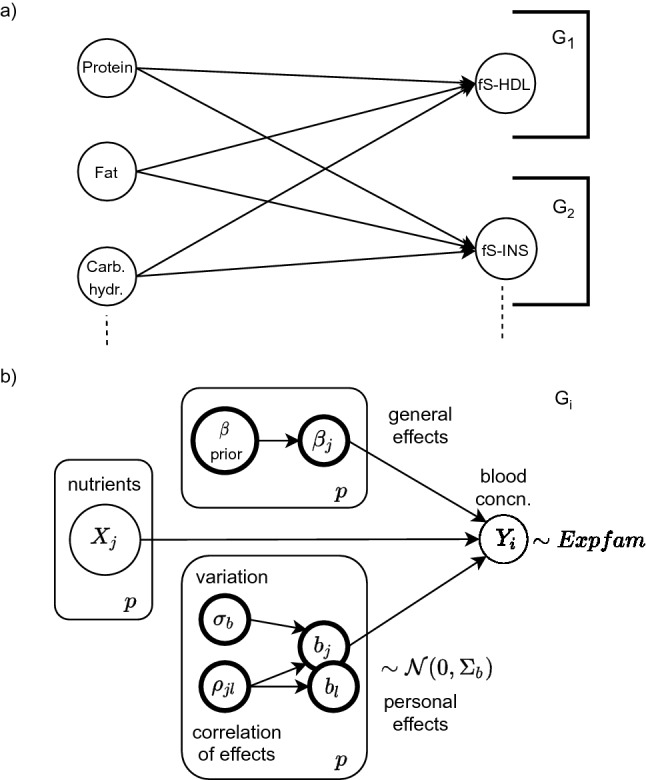
In (**a**) a bipartite Bayesian network is constructed from a few of the variables in Sysdimet data. The overall network factorizes into subnetworks $G_1$ and $G_2$ that form independently estimated local probability distributions. In (**b**) a general concentration-specific graphical model $G_i$ is shown. It includes *p* observed random variables *X* for the nutrient levels and the corresponding concentration level $Y_i$. The latent variables of each nutritional effect are estimated from the data. Personal variations ${\mathrm {b_i}}$ are assumed to follow Normal distribution, but blood concentrations can also be better modeled with Gamma distribution. Evaluation of the directed graphical model starts from the prior and the observed nodes. Their sampled values are propagated as input to downward nodes, and finally, the linear predictor (3) in the concentration node $Y_i$ gathers them all and results in an estimation.

The personal effects are calculated as follows by using the estimated matrices $\hat{\varvec{T}}_{ik}$ and $\hat{\varvec{L}}_{ik}$ that describe the effect behaviour

4 $$\begin{aligned} \begin{aligned} \varvec{z}&\sim \mathcal {N}(0, \varvec{I}) \\ \hat{\varvec{b}}_{ik}&= \hat{\varvec{T}}_{ik} \hat{\varvec{L}_{ik}} \varvec{z}, \end{aligned} \end{aligned}$$

where multiplying standard normal data $\varvec{z}$ with the learned Cholesky decomposition $\hat{\varvec{L}}_{ik}$ generates a multivariate distribution with the personally correlated effects of nutrients. Then, multiplying it with $\hat{\varvec{T}}_{ik}$ adds the estimated standard deviation of personal effects to the distribution. It should be noted that values in $\hat{\varvec{b}}_{ik}$ are only personal adjustments from the general effects $\hat{\varvec{\beta }}_i$ for subject *k* and the full personal effect is then their sum $\hat{\varvec{\beta }}_i + \hat{\varvec{b}}_{ik}$. The concentration-specific correlation matrix $\hat{\varvec{C}}_{ik} = \hat{\varvec{L}}_{ik} \hat{\varvec{L}}_{ik}'$ holds correlations $\rho _{jl}$ between each $j,l = 1,\dots ,p$ pair of personal effects (Fig. 2b). This matrix can be further studied for finding the reasoning of personal effect estimates.

Definition of the expected value is further extended to model the correlation of $n_k$ successive observations of a person *k* with a first order auto-regressive process, AR(1),

5 $$\begin{aligned} \begin{aligned} y_{ik}^{(t)} = \mu _{ik}^{(t)} + \rho _i(y_{ik}^{(t-1)}-\mu _{ik}^{(t-1)}) + \epsilon _t,\, t = 2,\ldots ,n_k, \end{aligned} \end{aligned}$$

where coefficient $\rho _i$ quantifies the correlation of successive observations of concentration $Y_i$, $y_{ik}^{(t-1)}$ is a concentration level from the previous personal measurement and $\epsilon _t$ is noise in the process.

The following sections elaborate considerations on network structure, choices for the personal effect model matrix $\varvec{Z}_{ik}$, shape of the local distribution *P*(.), and the prior distribution of general effect parameters $\varvec{\beta }$.

### Assumptions for the network structure

Only such networks where nutrients are affecting blood concentrations are considered plausible for modeling the effects of nutrients, and so a network prior $P(G_i) = 1$ is set for those networks, and $P(G_i) = 0$ for all the others in Eq. (1). This makes all the considered networks *bipartite* with two layers of random variables. The layers are assumed fully connected and so every nutrient is a possible predictor of any blood concentration. This structure is illustrated in Fig. 2a.

It is also assumed that all the effects from nutrients and other predictors may contain personal differences, and because of that, the inputs are same for both general and personal parts in Eq. (3) by assuming identical model matrices $pa_X(Y_{ik}) = pa_Z(Y_{ik})$. It is possible, though, to leave some of the effects common for all the subjects by dropping the corresponding column from $\varvec{Z}_{ik}$ if it is known that these effects cannot vary among persons.

### Distributions of random variables

Normal distribution is used as a starting assumption for all the observed random variables at the network. Reference intakes of nutrients are based on normal distribution^20^ and so it is practical to use the same distribution for dietary nutrient levels here as well. For the concentration values in Eq. (2) this means $Y_{ik} \sim \mathcal {N}\big (\varvec{\mu }_{ik}, \varvec{\Sigma }_{ik}\big )$ where $\varvec{\mu }_{ik}$ is a vector of expected values from Eq. (3) and $\varvec{\Sigma }_{ik}$ is a covariance matrix of the multivariate distribution.

The blood concentration values are non-negative and usually right-skewed with occasional values greatly above average. This kind of biometric data is usually better modeled with $\mathrm {Log-Normal}$ or $\mathrm {Gamma}$ distribution than with normal distribution^21^. In our model, $\mathrm {Gamma}$ distribution is considered as an alternative distribution candidate as it allows keeping the regression parameters additive by using an identity link function with $Y_i \sim \mathrm {Gamma}\Big (\alpha _i, \frac{\alpha _i}{g(\varvec{\mu }_i)}\Big )$ where $\alpha _i$ is a shape parameter of $\mathrm {Gamma}$ distribution and $g(\varvec{\mu }_i) = \varvec{\mu }_i$ is the identity link function for the expected value. Parameter additivity achieved with the identity link is important as it allows using the same parameter estimates in expected values of both normal and Gamma distributed random variables. This is valuable in future use cases where normally distributed nutrient levels are conditioned from estimated effects and given reference values of concentrations. Parameters are also easier to interpret in the additive scale.

### Choices of the prior distribution for the general effects

Parameters of the network are estimated with Bayesian regression. Before the estimation, all predictors are standardized on the same scale. This allows using the parameters $\beta _j$ and $b_j$ at the concentration-specific vectors $\varvec{\beta }_i$ and $\varvec{b}_{ik}$ in Eq. (3) as direct measures of effect strength. Bayesian modeling allows applying different prior distributions for parameters, and two different prior distributions are considered for the general parameters $\beta _j$. As a first option, when general effects are studied in detail, it is justifiable to use a vague prior that lets even the weakest of signals to emerge^22^. Here, Cauchy distribution is used with

6 $$\begin{aligned} \begin{aligned} \beta _0&\sim \mathrm {Cauchy(0,10)} \\ \beta _{j}&\sim \mathrm {Cauchy(0,2.5)}, j = 1,\ldots ,p, \end{aligned} \end{aligned}$$

by giving different scales for the intercept and the effect strengths as suggested by Gelman et al.^14^.

As a second option, when the effects of nutrition are predicted for new subjects, it might be better to use a shrinkage prior that forces weak signals to a minimum and allows the model to generalize better. Piironen and Vehtari^23^ give a thorough overview of Bayesian shrinkage priors and other model selection methods. For exploring a large number of possible predictors they suggest a projection method, but for this relatively small dataset a *regularized horseshoe prior* (RHS) is considered suitable. It allows more efficient computation and better predictive performance than the vague prior (6).

Regularized horseshoe prior for general coefficients in $\varvec{\beta }_i$ is defined with

7 $$\begin{aligned} \begin{aligned} \beta _j&\,|\,\lambda _j, \tau , c \sim \mathcal {N}\big (0, \tau ^2\tilde{\lambda ^2_j}\big ), \\&\tilde{\lambda ^2_j} = \frac{c^2\lambda ^2_j}{c^2+\tau ^2\lambda ^2_j}, \\&\lambda _j\sim \mathcal {C^+}(0,1), j=1, \ldots ,p, \end{aligned} \end{aligned}$$

where $\mathcal {C^+}$ denotes positive half-Cauchy distribution, $\lambda _j$ are local scaling parameters for parameters $\beta _j$, and $\tau$ is a global scaling parameter. Parameter *c* here is an additional regularization parameter, and without knowledge of ${\beta _j}$ parameter scales, a prior distribution suggested by Piironen and Vehtari^23^ is used, by setting $c^2 \sim \mathrm {Inv-Gamma}(\alpha , \beta ),\, \alpha = \nu / 2,\, \beta = \nu s^2 / 2$, where $\nu$ is degrees of freedom. Suggested value for $\nu$ is 1, but increasing it is reported to help possible computational problems.

Regularized horseshoe prior allows setting a hyperprior for the global shrinkage parameter $\tau$. This hyperprior $\tau _0$ allows applying domain knowledge about the number of effective predictors. It regularizes the highest coefficient values and lets the weaker signals to emerge despite the shrinkage. Regularized horseshoe still shrinks some weak signals that the vague prior allows but an optimal value for $\tau _0$ hyperprior makes it a good compromise for predictions. The value for optimal global shrinkage $\tau _0$ is calculated from the expected number of nonzero coefficients, $p_0$, with

8 $$\begin{aligned} \begin{aligned} \tau _0 = \frac{p_0}{p-p_0}\frac{\sigma }{\sqrt{n}}, \end{aligned} \end{aligned}$$

where *p* is the total number of predictors and $p_0$ is in this application the assumed number of nutrients affecting a blood concentration. Piironen and Vehtari^22^ show further that even a crude guess for number of nonzero parameters improves the prediction accuracy and computation time. With the Sysdimet dataset, it is approximated that one third of the nutrients might be significant for each measured concentration. This makes $\tau _0 = \frac{7}{22-7}\frac{1}{\sqrt{424}} \approx 0.023$.

Sensitivity analysis of the prior choice with the Sysdimet dataset is illustrated in Supplementary Table S4. It shows that estimation of the personal effect standard deviations $\hat{\sigma }_{bi}$ in Eq. (3) is not sensitive to the prior distribution of the general effects $\beta _j$. The prior choice affects mainly on how the personal effect is shared between the general and personal parts. Regularizing horseshoe prior puts less weight on generally weak effects and gives more weight to personal variations.

### Clustering of personal effects

Bayesian network factorizes the joint distribution of concentrations into distinct local distributions. For considering the overall reaction profiles, the personal effects from all the concentrations are pooled together and then clustered for finding self-similar groups of behavior. The clustering is not implemented as part of the model, but in the analysis of the model estimations for gaining more insights on the results. For this, standard k-means clustering^24^ is applied to the means of personal effect distributions, $\hat{\varvec{\beta }}_i + \hat{\varvec{b}}_{ik}$. It should be noted that in this way the clustering shows only average reactions within the clusters and more extreme personal differences can be found.

In k-means clustering, it is essential to find an optimal number of clusters. The number is approximated with the elbow method^25^ where a within-cluster sum of squares ratio is plotted for different numbers of clusters. The elbow point of the plot, where this sum of squares ratio stops decreasing substantially, shows the approximate number of clusters that are distinguishing clearly.

### Estimation of the Bayesian network

Probabilistic programming language *Stan*^26^ was used in implementing and estimating the local probability distributions $G_i$ of the Bayesian network as defined in Eq. (2). These blood concentration distributions were estimated with MCMC-sampling by using Stan’s Hamiltonian Monte Carlo (HMC) algorithm and the estimated distributions of parameters were collected as a joint Bayesian network *G* with a custom R-language code and iGraph^27^ R-package.

The result is a generative model that allows drawing samples of predicted blood concentrations when a dietary composition is given by using *ancestral sampling*^17^. In this method, the root nodes, including fixed hyperpriors of parameters and all the observed inputs, are populated first with values. Their values are then propagated downwards in the directed graph as inputs to the next connected variables until the linear predictors at the concentration variables are reached. Then, all the distribution parameters are estimated and predictions can be drawn. This flow is illustrated in Fig. 2b.

This ancestral sampling is used in posterior predictive checking^14,19,28^ to evaluate the model fit. The original input data are fed to the graphical model and, if the parameters are estimated correctly, the outcome is expected to match closely the true concentrations. Both visual posterior predictive checks and numerical error metrics are used to detect biased estimations that suggest refining the model structure.

## Data application

The proposed mixed-effect Bayesian network (MEBN) model was applied to personal nutrition data from the Sysdimet study. The aim of this application was to gain statistical information about general nutritional effects in these data, reveal personal variations from the general effects, and finally predict personal networks of the nutritional effects. This model estimation and the following analysis can be fully replicated with data and code notebook at the Supplementary Information.

### Evaluation of the model performance

Several model candidates were developed with alternative distributions of concentrations, different prior distributions of general effects, and with and without an autocorrelation structure. These options were described in the method section. The model candidates were compared visually with posterior predictive checks and numerically with mean squared errors. The plots of visual posterior checks for different model candidates are available online in Supplementary Figs. S3, S4, S5, S6, and S7. The used numerical normalized root mean squared error metrics are defined with

9 $${\text{Network}}\;{\text{NRMSE}} = \frac{{\sum\limits_{{i = 1}}^{v} {NRMSE_{{G_{i} }} } }}{v},\;\;\;{\text{NRMSE}}_{{{\text{G}}_{{\text{i}}} }} = \frac{{\sqrt {\frac{1}{n}\Sigma _{{j = 1}}^{n} (\hat{y}_{{i_{j} }} - y_{{i_{j} }} )^{2} } }}{{\bar{y}}},$$

where the error metrics for the whole network is calculated as mean of errors from local distributions $G_i, i = 1, \ldots ,v$. Normalization in the error metrics allows comparing the predictions of the different concentrations on the same scale.

The best overall model fit was reached with a model version that uses normal distribution for insulin concentration (fsins) and Gamma distribution for all the other concentrations. This version is called EXPFAM AR(1)-model as the model consists of several different distributions from the exponential family and it had network NRMSE of 0.07. It uses the autocorrelation process of successive observations from Eq. (5) that also improved the model fit. Using regularized horseshoe shrinkage prior in Eq. (7) for the general effects provided a simpler model with only a slight increase in the network NRMSE to 0.07. As the shrinkage prior provides a more general model, and also faster and more stable computation, it is used in predictions at the model cross-validation. A detailed comparison of different model versions is shown in Table 1 and is illustrated in Supplementary Fig. S8.

**Table 1:** Comparison of model candidates.

Model	NRMSE					
	HDL-chol.	LDL-chol.	Insulin	Total chol.	Glucose	BN Mean
Normal	0.04	0.06	0.22	0.06	0.06	0.09
Gamma	0.02	0.06	0.28	0.05	0.03	0.09
Gamma AR(1)	0.01	0.03	0.27	0.04	0.03	0.07
Gamma AR(1) RHS	0.01	0.03	0.29	0.04	0.03	0.08
EXPFAM AR(1)	0.01	0.03	0.23	0.04	0.03	0.07
EXPFAM AR(1) RHS	0.01	0.03	0.23	0.04	0.03	0.07
Gamma AR(1) RHS CV	0.02	0.05	0.44	0.06	0.05	0.12
EXPFAM AR(1) RHS CV	0.02	0.05	0.45	0.06	0.05	0.13

Model candidates were compared with a normalized root mean squared error (NRMSE) separately for each concentration and with mean accuracy of the whole Bayesian network. The final versions (Gamma AR(1) RHS CV and Expfam AR(1) RHS CV) were evaluated with cross-validation (CV).

In predicting the new and unseen concentration values with cross-validation^14,19^, we used the available personal reaction history and the information from other persons in estimating the prediction model. The model assumes with $\mathrm {AR(1)}$ autocorrelation that the blood concentration level is correlated only with one previous concentration measurement in addition to the previous food record. We also assume that personal reactions to nutrients are similar in every week of the study. This allows using 3 $\times$ 10-fold validation by splitting the concentration time series of weeks 0, 4, 8, and 12 in Fig. 1 into three distinct parts where the true values from weeks 4, 8, and 12 were held out from the model estimation for a tenth of the subjects at a time. The estimated model was then used for predicting the concentrations for these held out subjects. The week 0 was used only as an input for the week 4 prediction. Network NRMSE increased then from in-sample error 0.07–0.13 for out-of-sample predictions of cross-validated (CV) models.

Yet, NRMSE metrics do not say much about the actual usefulness of the model. In personal nutritional guidance, it is essential to know how a given diet will affect the future blood concentrations, or any other personal goal that is measured. Will the concentrations increase or decrease towards their reference values? For better understanding, a custom classification test was used to evaluate if the model predicts correctly the direction of change in the blood concentrations by using the same cross-validation folding of the data. In this classification, we predict only if the concentration increases or decreases from its past value without considering the actual level of the concentration.

The classification test enables us to compare the Bayesian network to other classification methods. Extreme Gradient Boosting (XGBoost)^29^ method was chosen for comparison as it is a well-performing decision tree method and similar to the computational approach in glucose prediction of Zeevi et al.^6^. XGBoost is a non-parametric machine learning method that requires separate training and testing partitions of data. Cross-validation was used also with XGBoost to ensure that any specific data partitioning to training and test sets do not affect the result. XGBoost can be sensitive to predefined hyperparameter values and to mitigate this a hyperparameter tuning was used to find the best performing values. Similarly to MEBN, the direction of the next blood concentration was predicted based on previous food records and one last concentration measurement.

In the comparison shown in Table 2 and Supplementary Fig. S9, our MEBN model repeats the increases and decreases of the in-sample measurements with 86% average accuracy for insulin concentration and over 80% for HDL- and LDL-cholesterol concentrations. Plasma glucose was most difficult to repeat with nutrient predictors only, and there 72% accuracy was reached. Plasma glucose can also react to other factors like the amount of exercise, time from last meal, and inflammation. When regularized horseshoe shrinkage prior from Eq. (7) was used for the general effects, the accuracy dropped a few percent overall with a similar trend. The cross-validated comparison between XGBoost and MEBN shows them to be comparable with similar classification accuracy ranging from 60 to 70%. Supplementary Information includes also a similar comparison with a Random Forest decision tree^30^ that uses bagging instead of boosting to improve the performance. The predictive accuracy of Random Forest is found similar to XGBoost and MEBN with a similar trend in concentration specific accuracy.

**Table 2:** Comparison of modeling methods.

Method	Prediction accuracy for the direction of change				
	HDL-chol.	LDL-chol.	Insulin	Glucose	Total chol.
MEBN	81%	85%	85%	74%	83%
MEBN RHS	78%	81%	84%	70%	80%
MEBN RHS CV	63%	67%	68%	59%	64%
XGBoost CV	61%	69%	68%	60%	71%

Comparison of modeling methods in a classification test where the direction of change for future concentration is predicted based on its past value and past diet. In-sample accuracy of mixed-effect Bayesian network (MEBN) assures that the found personal effects are real in this sample. The cross-validated (CV) accuracy is lower for out-of-sample observations but it is similar to the compared XGBoost decision tree method.

### General effects of nutrition and their personal variation

**Figure 3:**
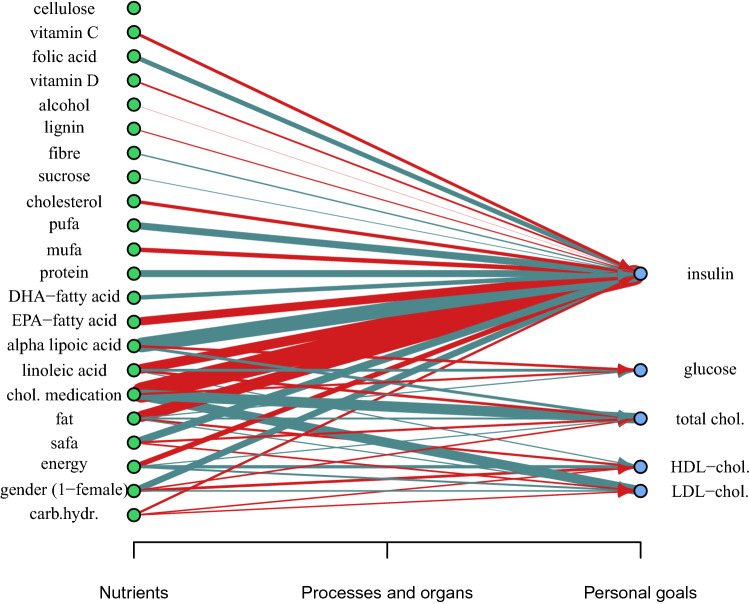
Visualization of the mixed-effect Bayesian network shows the general effects from nutrients ($j=1,\dots ,22$) to concentrations ($i=1,\dots ,5$). The strength of the effects from parameters $\hat{\beta }_{ij}$ are indicated by the thickness of the edge. Red edges denote the increasing effect and blue edges denote the decreasing effect. The Bayesian network corresponds to the systems biology of personalized nutrition by connecting the statistical effects of nutrients to personal goals of well-being. The goal might be, for example, reaching the reference values of the blood concentrations. The figure is plotted with iGraph package for R language (v 1.2.6, https://igraph.org/r).

Visualization in Fig. 3 shows the constructed bipartite Bayesian network *G* that is based on the most accurate EXPFAM AR(1) model version with in-sample predictions. The visualization shows the general effects of nutrients by indicating the means of $\hat{\beta }_{ij}$ parameter distributions with the thickness of the edges between a concentration *i* and its contributing nutrients *j*. The visualized model uses vague priors from Eq. (6) on parameters $\beta _{ij}$ for emphasizing the general effects and putting less weight on personal variations. This graph visualization offers an overview of the most considerable general effects. Examples of personal differences can be seen by comparing this general graph to personal graphs in Fig. 5.

**Figure 4:**
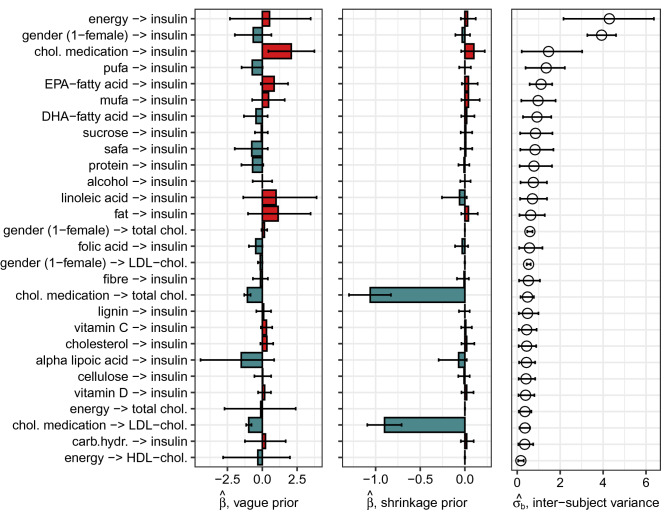
List of nutritional effects where the effect is either generally strong or it has high variation between subjects. If a generally insignificant effect, with low $\hat{\beta }_{ij}$, has a high inter-subject variation $\hat{\sigma }_b$, it can still be significant for someone. The figure shows posterior means with 90%-credible intervals and compares the effect of shrinkage prior (7) to vague prior (6) in $\hat{\beta }_{ij}$ estimation. The figure is plotted with ggplot2 package for R language (v 3.3.2, https://ggplot2.tidyverse.org).

More detailed information on the effects and their behavior is given in Table 3 and Fig. 4. The figure compares $\hat{\beta }_{ij}$ estimations with vague (6) and shrinkage (7) prior distributions. As expected, the cholesterol-lowering medication has a strong decreasing effect on serum cholesterol concentrations, but it also increases insulin concentration. The effect is so strong that it overwhelms the most nutritional effects of the model.

**Table 3:** Those effects of nutrition that have highest variations between the studied subjects.

Nutrient or feature	Concentration	Inter-subject	Personal effect ($\hat{\beta }_{ij} + \hat{b}_{ijk}$)	
		Variation ($\hat{\sigma }_{b_{ij}}$)	Min.	Max.
Energy	Insulin	4.29 [2.16; 6.37]	0.12 [− 4.00; 4.43]	1.12 [− 3.00; 6.09]
Gender (1-female)	Insulin	3.93 [3.26; 4.60]	− 1.03 [− 4.82; 1.64]	0.19 [− 2.59; 5.56]
Chol. medication	Insulin	1.46 [0.21; 3.02]	− 1.29 [− 7.50; 3.70]	8.63 [2.18; 15.94]
Pufa	Insulin	1.35 [0.39; 2.22]	− 1.12 [− 3.47; 0.29]	− 0.40 [− 1.81; 1.59]
EPA-fatty acid	Insulin	1.11 [0.58; 1.63]	0.23 [− 1.06; 1.61]	1.72 [− 0.14; 4.80]
Mufa	Insulin	0.97 [0.19; 1.79]	− 0.96 [− 3.83; 1.29]	2.27 [− 0.51; 6.01]
DHA-fatty acid	Insulin	0.92 [0.27; 1.59]	− 0.62 [− 2.28; 0.72]	− 0.10 [− 1.47; 1.90]
Sucrose	Insulin	0.85 [0.14; 1.64]	− 0.28 [− 1.83; 0.63]	0.12 [− 0.75; 1.82]
Safa	Insulin	0.83 [0.14; 1.69]	− 1.46 [− 4.22; 0.48]	0.30 [− 2.09; 4.13]
Protein	Insulin	0.78 [0.11; 1.62]	− 0.97 [− 2.93; 0.42]	− 0.40 [− 1.88; 2.06]
Alcohol	Insulin	0.75 [0.16; 1.39]	− 0.17 [− 1.49; 0.89]	0.11 [− 0.98; 1.48]
Linoleic acid	Insulin	0.71 [0.13; 1.39]	0.18 [− 2.84; 4.95]	0.91 [− 1.81; 5.68]
Fat	Insulin	0.63 [0.11; 1.28]	0.70 [− 2.75; 4.28]	1.73 [− 1.37; 6.26]
Gender (1-female)	Total chol.	0.59 [0.47; 0.72]	− 0.70 [− 1.98; 0.24]	0.82 [0.01; 1.94]
Folic acid	Insulin	0.57 [0.09; 1.18]	− 0.66 [− 2.22; 0.27]	− 0.22 [− 1.15; 1.60]
Gender (1-female)	LDL-chol.	0.53 [0.44; 0.63]	− 0.67 [− 1.63; − 0.02]	0.36 [− 0.27; 1.36]
Fibre	Insulin	0.52 [0.08; 1.06]	− 0.24 [− 1.37; 0.70]	0.00 [− 0.91; 1.23]
Chol. medication	Total chol.	0.48 [0.16; 0.78]	− 1.39 [− 2.50; − 0.83]	− 0.71 [− 1.32; 0.50]
Lignin	Insulin	0.48 [0.08; 0.99]	− 0.40 [− 2.75; 0.85]	0.68 [− 0.43; 2.48]
Vitamin C	Insulin	0.44 [0.07; 0.90]	− 0.76 [− 2.67; 0.65]	2.43 [0.43; 4.82]
Cholesterol	Insulin	0.44 [0.06; 0.88]	0.27 [− 0.75; 1.13]	0.39 [− 0.47; 1.48]
Alpha lipoic acid	Insulin	0.43 [0.09; 0.83]	− 1.69 [− 6.65; 1.19]	− 0.86 [− 5.63; 1.98]
Cellulose	Insulin	0.41 [0.07; 0.84]	− 0.12 [− 1.76; 0.96]	0.23 [− 0.94; 1.86]
Vitamin D	Insulin	0.39 [0.06; 0.80]	− 0.04 [− 2.16; 1.02]	0.52 [− 0.32; 1.97]
Chol. medication	LDL-chol.	0.36 [0.11; 0.60]	− 1.08 [− 1.77; − 0.70]	− 0.81 [− 1.23; 0.12]
Energy	Total chol.	0.36 [0.07; 0.66]	− 0.18 [− 3.76; 3.27]	− 0.07 [− 3.67; 3.36]
Carb.hydr.	Insulin	0.35 [0.05; 0.74]	− 0.29 [− 3.11; 1.96]	1.00 [− 1.44; 4.29]
Gender (1-female)	Glucose	0.30 [0.24; 0.36]	− 0.10 [− 0.48; 0.12]	0.00 [− 0.22; 0.38]
Gender (1-female)	HDL-chol.	0.24 [0.20; 0.29]	0.09 [− 0.29; 0.34]	0.53 [0.21; 0.96]
Energy	Glucose	0.23 [0.05; 0.41]	− 0.06 [− 3.32; 3.20]	0.06 [− 3.17; 3.35]
Energy	LDL-chol.	0.23 [0.04; 0.47]	− 0.20 [− 3.53; 3.03]	− 0.12 [− 3.45; 3.10]
Chol. medication	HDL-chol.	0.18 [0.07; 0.29]	− 0.19 [− 0.54; 0.02]	0.08 [− 0.17; 0.49]
Energy	HDL-chol.	0.17 [0.04; 0.30]	− 0.29 [− 3.63; 2.77]	− 0.24 [− 3.60; 2.77]
Cellulose	LDL-chol.	0.14 [0.06; 0.21]	− 0.03 [− 0.17; 0.08]	0.00 [− 0.11; 0.15]
Cellulose	Total chol.	0.13 [0.03; 0.22]	− 0.02 [− 0.17; 0.12]	0.00 [− 0.13; 0.16]
Sucrose	Glucose	0.12 [0.04; 0.20]	− 0.06 [− 0.27; 0.05]	− 0.01 [− 0.11; 0.17]
Chol. medication	Glucose	0.12 [0.02; 0.24]	0.04 [− 0.52; 0.36]	0.39 [0.06; 0.96]
Sucrose	Total chol.	0.12 [0.02; 0.22]	− 0.01 [− 0.29; 0.13]	0.06 [− 0.05; 0.22]

These 38 (of all 110) effects from nutrients and other modeled features $(j = 1,\dots ,22)$ to blood concentrations $(i = 1,\dots ,5)$ had the highest variations between the studied subjects (k = 1,...,106). The estimated standard deviation ($\hat{\sigma }_{b_{ij}}$) of personal effects varies from 4.29 to 0.02, and this table is sorted to include the most varying effects with the standard deviation over 0.10. The table also shows the minimum and maximum of the personally adjusted effects $(\hat{\beta }_{ij} + \hat{b}_{ijk})$ with 90%-credible interval.

Besides the general effects, our interest is on the variations of effects between the subjects. The amount of effect variations are estimated in standard deviation parameters $\hat{\sigma }_{b_i}^{(1)},\dots ,{\hat{\sigma }_{b_i}^{(p)}}$ of Eq. (3). The highest variations are found at the effects of energy intake and gender to insulin concentration. On these effects, 90%-credible intervals of standard deviance estimations are far from zero. In the contributing nutrients, the effect of polyunsaturated fatty acid (PUFA) and eicosapentaenoic acid (EPA) have also variation between subjects on insulin concentration, but it is less certain. Details of most varying effects are seen in Table 3 that lists 38 of the all 110 analysed effects that have highest standard deviations of personal effects. Table also shows the minimum and maximum of those effects from all the subjects. We also analysed if the found reactions are personally unique or do they form self-similar groups.

### Hidden clusters of personal effects

We applied k-means clustering^24^ to personal effects ($\hat{\beta }_{ij} + \hat{b}_{ijk}$) first with all the predictors and then with nutrient predictors only. In both cases the elbow plots^25^ revealed four clusters that had distinguishing differences between the effects. The elbow plots are available in Supplementary Figs. S13 and S15.

When all the predictors were considered, the subjects grouped into clusters with 83 (78.3%), 11 (10.4%), 5 (4.7%), and 7 (6.6%) of all the 106 subjects. The clusters showed clearly the personal variations in the effects of cholesterol medication that was also seen in Fig. 4. The cholesterol medication decreases cholesterol concentrations in all clusters but increases the serum insulin concentration only with part of the subjects. In the second cluster with 11 of 106 subjects (10.4%) the medication has no effect on insulin concentration, but for 7 subjects (6.6%) it increases insulin concentrations clearly and for the rest of the subjects the effect is only slightly increasing. This effect of cholesterol-lowering medication increasing insulin concentrations has also been shown in a clinical trial^31^. These clusters are illustrated in detail in Supplementary Fig. S14.

As the effect of cholesterol medication dominates the clusters, and our main interest is to find personal differences especially in nutritional effects, another clustering was considered with nutrient predictors only. This time the subjects were also grouped into four clusters with 18 (17%), 36 (34%), 4 (3.8%), and 48 (45.3%) of all 106 subjects. The second and fourth clusters with most of the subjects are quite similar to the general behaviour of insulin reactions as seen in Figs. 3 and 4. Distinguishing differences are found in the first and third clusters. The first cluster, with 18 subjects, is characterized by slight insulin decreasing effects of monounsaturated fatty acids (MUFA) and vitamin C. However, in the third cluster, with only 4 subjects, those effects are opposite and clearly increasing. In addition, with these subjects lignin and vitamin D are slightly decreasing the insulin concentration while this effect is opposite and increasing in other clusters. These cluster characteristics are illustrated in Supplementary Fig. S16. This clustering analysis considers only typical behaviour within the clusters, and to conclude the analysis, two different personal reaction types are compared in detail and possible personal dietary guidance is discussed.

### Personally predicted reaction types

The clustering revealed two different reaction types for serum insulin concentration. In one group the insulin concentration raises rapidly and in the other groups, the response is weaker or average. Constantly high concentrations of serum insulin may lead to insulin resistance and personal guidance for lowering the concentration would be necessary. What nutrients cause these differences and what personal recommendations can be given?

**Figure 5:**
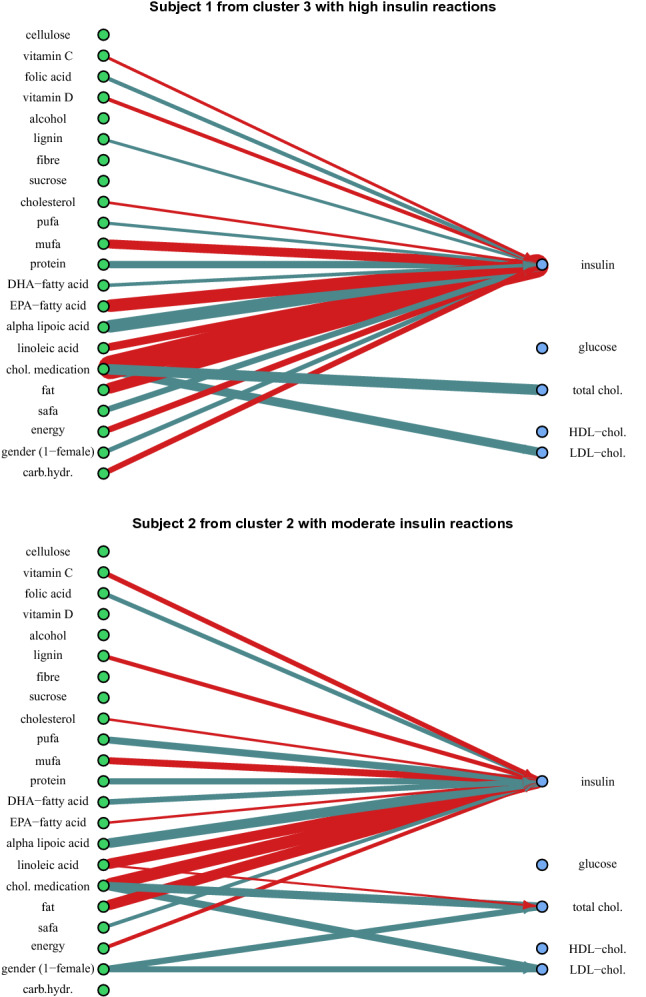
Two subjects were picked from differently reacting clusters and their personally predicted reaction graphs show differences from the general behaviour in Fig. 3 and with each other. The visualization shows 20 most personally prominent effects ($\hat{\beta }_{ij} + \hat{b}_{ijk}, i=1,\dots \,5, j=1,\dots ,22, k=1,2$). The red edges denote increasing effect and the blue edges denote a decreasing effect. Subject 1 is reacting generally with a greater increase in insulin concentration than subject 2, but there are even contradicting effects between the subjects. Vitamin D increases insulin concentration for subject 1 only and the effect of lignin is small but opposite between the subjects. The figure is plotted with iGraph package for R language (v 1.2.6, https://igraph.org/r).

Two subjects are picked from these differently reacting clusters and their personally predicted reaction types are compared in Fig. 5. The biggest differences between these groups are found in reactions of insulin concentration to saturated fatty acids (SAFA), monounsaturated fatty acids (MUFA), and vitamin C. During the study, the average personal serum insulin concentration for the subject 1 was 29.38 mU/l and 22.04 mU/l for the subject 2. Both of these concentrations are high in comparison to the reference values, but there are personal differences in the nutrients that affect these concentrations. The personal averages of all the concentrations and the following personal effects can be found in Supplementary Tables S8 and S9.

In general, subject 1 reacts to most nutrients with increasing serum insulin concentration and the overall energy content of the diet has a greater effect on the concentration with slope 1.26 (90% credible interval, CI [− 1.96; 4.79]). For the subject 2, the effect of the energy content is lower with slope 0.67 (90% CI [− 2.47; 3.98]).

Specific personal differences are found in reactions to saturated fat, lignin, vitamins C and D, and eicosapentaenoic acid (EPA) fatty acid. Saturated fat decreases serum insulin concentration for subject 2 with a slope − 1.02 (90% CI [− 2.84; 0.82]) but for subject 1 the effect mean is close to zero. Then again, the subject 1 has a greater lowering effect from lignin with − 0.44 (90% CI [− 2.05; 0.83]) while the same for the subject 2 is − 0.1 (90% CI [− 1.39; 1.06]).

The subjects have yet different nutrients that specifically increase the serum insulin concentration. For the subject 1, vitamin C has an increasing effect of 2.36 (90% CI [0.80; 3.80]) while it decreases the concentration with a slope − 0.66 (90% CI [− 2.32; 0.82]) for subject 2. Vitamin D has also a similar effect. On the other hand, EPA increases insulin concentration for subject 2 with a slope 1.95 (90% CI [0.20; 4.03]) while the same effect is only 0.15 (90% CI [− 1.79; 1.82]) for subject 1. With these findings, it would seem reasonable to give these subjects different nutritional guiding for lowering their insulin concentrations.

## Discussion

In this work, we have implemented a statistical model for the personal effects of nutrition. As our long-term goal is computing personalized nutrient intake recommendations for personalized nutrition, we aimed for a model that is biologically interpretable and has rich possibilities for inference. This was achieved with a Bayesian network that follows the systems biology of personalized nutrition^3^ from dietary nutrients to their biological response. The mixed-effect parameterization of our Bayesian network allows studying the effects in both general and personal levels. In addition to producing these estimates in an additive scale with full posterior distributions, our model also reveals the autocorrelation of successive concentration levels and the correlation between the effects of different nutrients. We argue that a high inter-individual variation in effects indicates a possibility, and perhaps a need, for personalized nutritional guidance.

We have confirmed that the learned effects between the nutrients and the concentrations match the existing clinical knowledge. As the in-sample classification performance and the posterior predictive check for the final model version are accurate, we are confident that the found personal variations are real and the method is able to reveal them. Still, the cross-validated accuracy of this model is not high enough for clinical applications for new patients. More data need to be collected to improve the predictive accuracy.

Our findings from the analyzed nutrition data reveals personal differences from the general behavior that might even be opposite between individuals. One example is the effect of monounsaturated fatty acid (MUFA) to insulin concentrations. The effect is generally increasing but in one group that contains 17% of the studied subjects, the effect is non-existing or even slightly decreasing. Also, a known side effect of the cholesterol-lowering medication is the increase of plasma insulin concentration^31^. Yet, this is not true for everyone. We have recognized clusters of subjects where the insulin increasing effect varied from considerable to non-existing.

In general, the clustering of personal effects showed that they are not unique, but tend to form self-similar clusters. There are studies that connect the differences in personal reactions to similar genotypes, like fatty acid desaturase 1 (FADS1) genotype affecting low-grade inflammation by linoleic acid^32^. On the other hand, Berry et al. report that even for identical twins the reactions are not identical^33^. They show that genetic factors explain only a part of the personal variation in responses at glucose, insulin, and triglycerides. Only a weak correlation was found between an individual’s genotype and responses to total fat and carbohydrates. Rest of the variation may be explained with lifestyle and other personal factors like gut microbiome.

In our future work, we are improving the model and building the inference for personalized nutrition. In comparison to the existing decision tree methods, our method reveals probability distributions for the personal contributions of the nutrients. This allows predicting the optimal amounts of different nutrients in personal diets that most probably take the concentrations towards their recommended levels. The reasoning for these predictions can also be justified by studying the learned personal effects correlation matrices $\varvec{\hat{C}_{ik}}$ in linear predictor Eq. (3). The mixed-effect models can be calibrated for new patients with only few observations when well-predicting model exists^34^. The calibrated model could be used in accurate personal concentration predictions, but it does not explicitly show the contributing nutrients for a new patient. Our model could be improved by analyzing the non-linearity of responses instead of the current linear predictor. This could give more information on time-varying intensity the nutrients affect the blood concentrations. Modeling the effect non-linearity would require more measurements from each subject than was available in our current data. In addition, the blood concentrations are now independently modeled, but studying the correlations between them is also possible by introducing new latent variables.

Our goal for personalized nutrition is to provide a rich diet while mitigating the personal health issues and risks of non-communicable diseases. As shown in this analysis, the causes for high insulin levels may vary between individuals from different nutrients to use of cholesterol-lowering medication, and differences like this should be taken into account at the nutritional guidance. We have estimated the personal reactions mainly from past diet and concentration history. As the causal mechanisms of these personal differences are studied and found, the Bayesian network can be extended to model them explicitly with new random variables. If the lifestyle and environmental factors affect the personal reactions more than genotypes^33^, it means that the personal reaction type can change over time. This also prefers our Bayesian estimation that can be updated with new information while having the previous model as a prior knowledge^17^.

## Supplementary Information

Supplementary Information.

## Data Availability

All data generated or analysed during this study are included in this published article (and its Supplementary Information files).
