# Supplementary material for: Mixed-effect Bayesian network reveals personal effects of nutrition
Source: Sci Rep. 2021 Jun 8;11:12016. doi: 10.1038/s41598-021-91437-3 (PMC8187367; doi:10.1038/s41598-021-91437-3)
Supplement: Supplementary file 1 — Supplementary Information. [file 41598_2021_91437_MOESM1_ESM.pdf]

Mixed-Effect Bayesian Network Reveals Personal Effects of Nutrition

Supplementary Materials

JARI TURKIA [1,2] LAURI MEHTÄTALO [1,3] URSULA SCHWAB [4,5]
VILLE HAUTAMÄKI [1]
[1] School of Computing, University of Eastern Finland, 80101 Joensuu, Finland
[2] CGI Suomi, Joensuu, Finland
[3] Natural Resources Institute Finland (Luke), Bioeconomy and Environment Unit, Yliopistokatu 6, 80101 Joensuu, Finland
[4] School of Medicine, Institute of Public Health and Clinical Nutrition, University of Eastern Finland, Kuopio, Finland
[5] Department of Medicine, Endocrinology and Clinical Nutrition, Kuopio University Hospital, Kuopio, Finland

Abstract

This notebook is supplementary material for the article "Mixed-Effect Bayesian Network Reveals Personal Effects of Nutrition". The notebook shows in detail how mixed-effect Bayesian network is constructed and how it is used to model the effects of nutrition. The hierarchical structure of the network model allows studying the effects in both typical and personal levels. In addition, it is shown that the personal effects form clusters of similarly behaving subjects. Finally, personal networks are predicted for unseen subjects with cross-validation. This shows that there exist personal differences in nutrional effects, and they can be detected from repeated observations of food records and blood concentrations.

In this work we propose Bayesian network as an appealing way to model and predict the effects of nutrition. Bayesian networks are directed graphical models that describe a joint probability distribution where nodes of the graph are random variables and edges between the nodes form a conditional probability structure. In nutritional modeling we assign nutritional composition of subjects' diet and different blood concentrations as those random variables, and study the connections between them. The goal is then to find such connections for the graph that most probably describe the correct conditional relationships between nutrients and their effects.

Personal nutrition data

We analyze a dataset from the Sysdimet study (Lankinen et al. 2011) that contains four repeated measurements of 17 nutrients, some basic information about the subject (gender, medication) and their corresponding blood concentrations, from 106 individuals.

Following spaghetti plots show the progress of blood concentrations during the 12-week study. Measurements of only ten from 106 subjects are shown at the plots for clarity. Our goal is to predict next personal blood concentration when subject's diet from past weeks is known.

When the future direction of the blood concentrations can be predicted, the typical and personal importance of nutrients contributing this change be estimated from the model parameters.

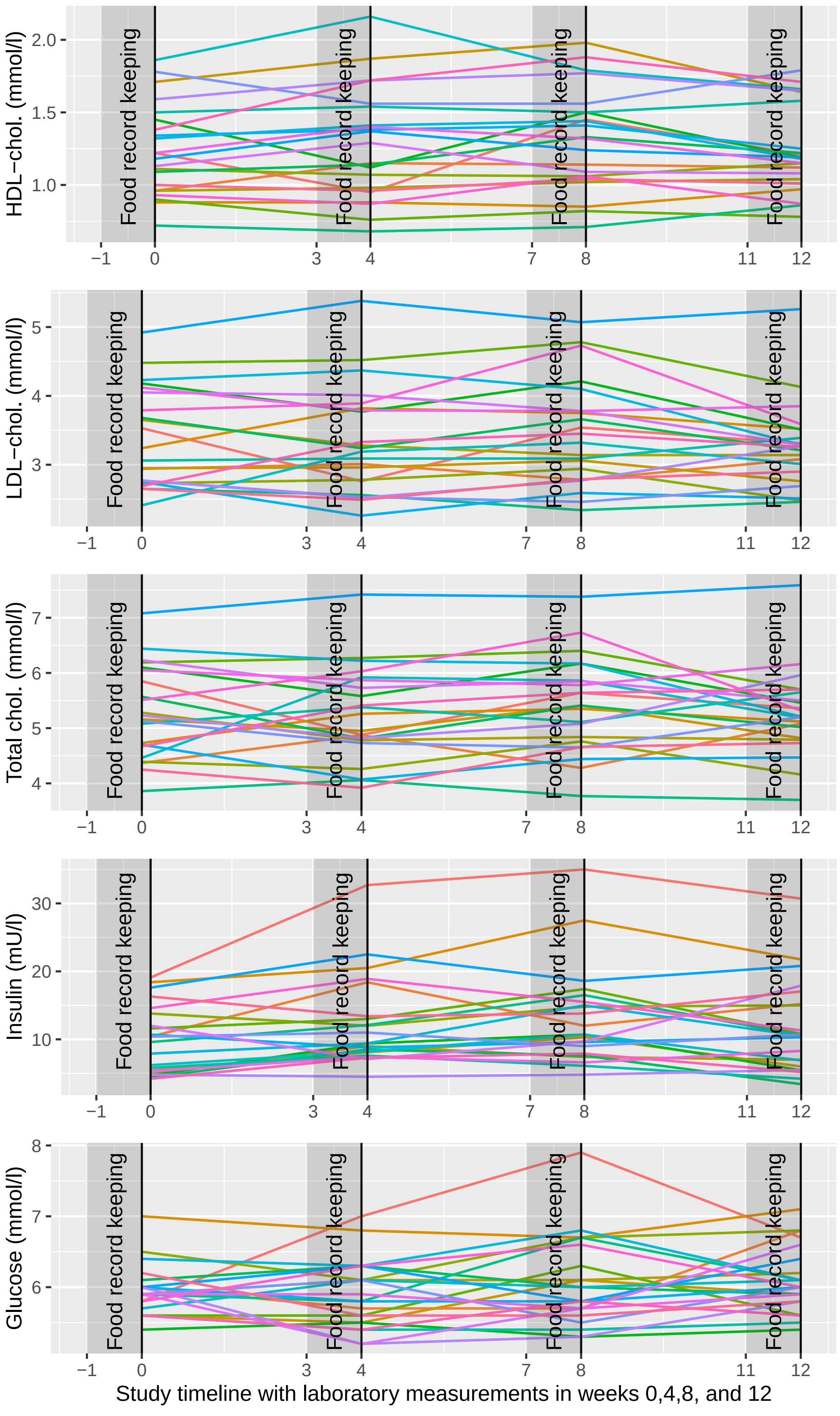

Supplementary Figure S 1: Progress of concentration levels during the 12-week study. Measurements were taken at weeks 0, 4, 8, and 12, and the food records were kept during the week before the measurement. The figure is plotted with ggplot2 package for R language (v 3.3.2, https://ggplot2.tidyverse.org).

Mixed-effect Bayesian network

We focus only on two-level, bipartite, graphs where nutrients and personal details are assumed to affect blood concentrations, and only the magnitude of the effect is left to be estimated.

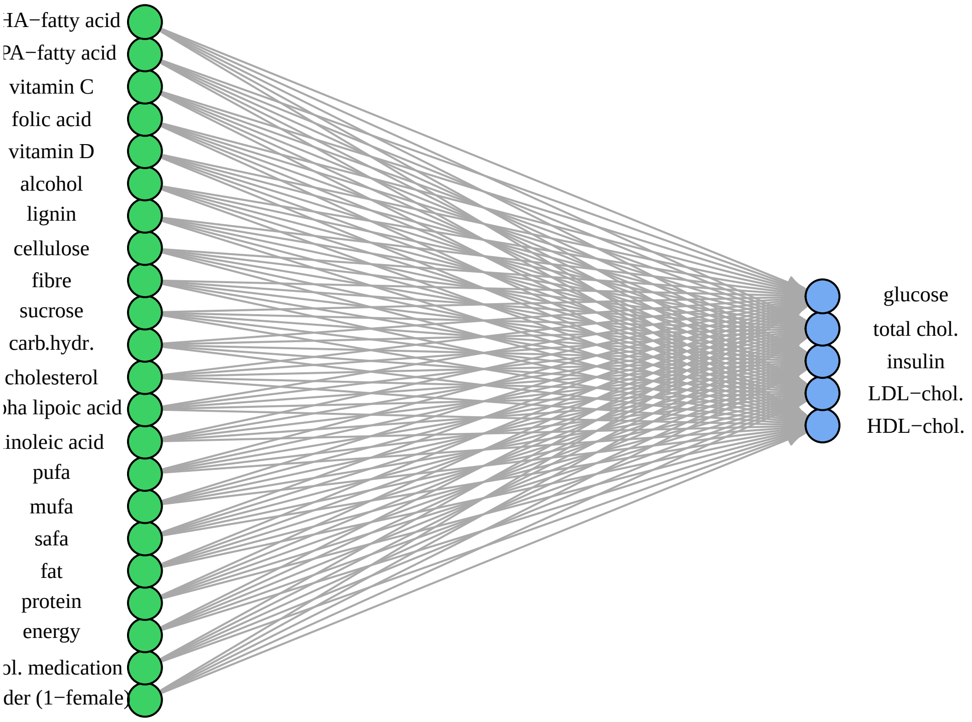

Supplementary Figure S 2: Initial, bi-partite, network structure. The figure is plotted with iGraph package for R language (v 1.2.6, https://igraph.org/r).

We assume a connection from every nutrient and personal information to every blood concentration value at the dataset. The model search is then focused on to estimating optimal coefficients describing the connections.

Model development

Our starting assumption is that both nutrients and the blood concentrations are normally distributed. This assumption may be naive as it allows the blood concentrations to have negative values. We normalize all the input values to same scale, so that the estimated regression coefficients can be used as a indicators of connection strenght.

```
sysdimet_normal <- mebn.bipartite_model(reaction_graph = initial_graph,
                                        inputdata = sysdimet,
                                        predictor_columns = assumedpredictors,
                                        assumed_targets = assumedtargets,
                                        group_column = "SUBJECT_ID",
                                        local_estimation = mebn.sampling,
                                        local_model_cache = "models/BLMM_normal",
                                        stan_model_file = "mebn/BLMM_normal.stan",
                                        normalize_values = TRUE)
```

```
## [1] "fshdl"
## [1] "fsldl"
## [1] "fsins"
## [1] "fskol"
## [1] "fpgluk"
```

Here we evaluate the fit of model candidates visually by plotting the posterior predictive distributions (PPC) over the distributions of the true blood concentrations. As expected, normal distribution in random variables is not fitting well. Normal distribution allows non-zero probability for negative blood concentrations resulting the probability to leak for impossible values. Normal distribution is also symmetric while the distributions of blood concentrations are usually more skewed to right.

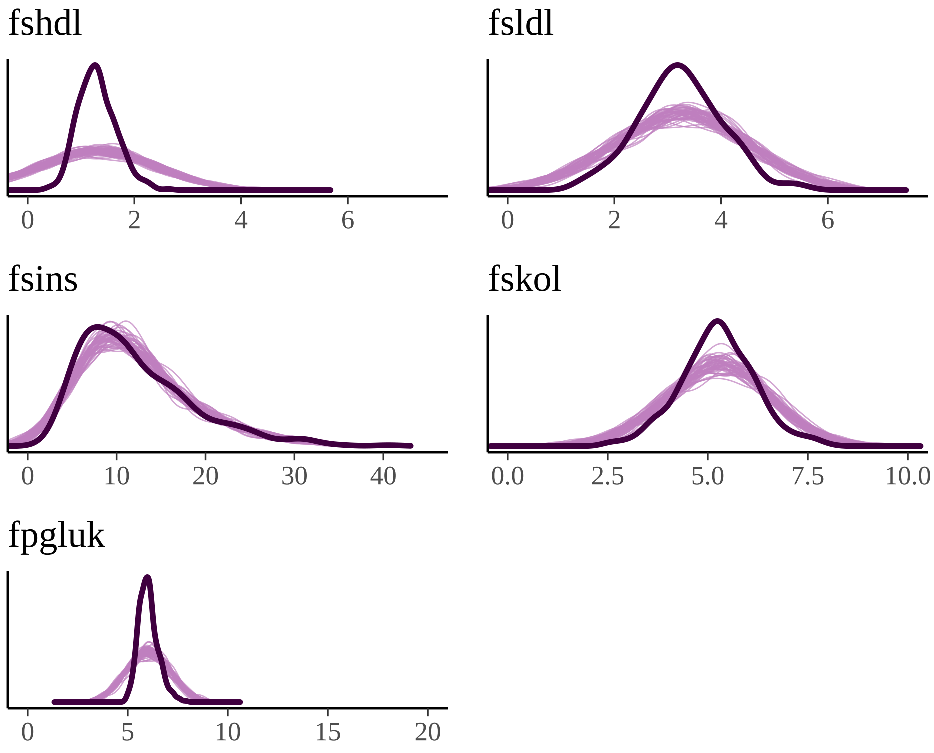

Supplementary Figure S 3: Visual posterior predictive check of local distributions with normal distribution. The figure is plotted with bayesplot package for R language (v 1.7.3, https://mc-stan.org/bayesplot).

Developing the model beyond normal distributions

More realistic probability distribution for blood concentrations would be Log-Normal or Gamma distributions (Limpert 2011). They both allow only positive values and model better the right tail of individual larger values. For further development, we choose Gamma distribution with identity link function. This is important as it keeps the regression coefficients of the nutrients on the same absolute scale than with Normal models. The regression coefficients are used as weights of the edges at the Bayesian network and they all should be on the same scale regardless of the random variables' distribution.

```
initial_gamma_graph <- mebn.new_graph_with_randomvariables(datadesc)

sysdimet_gamma <- mebn.bipartite_model(reaction_graph = initial_gamma_graph,
```

```
                        inputdata = sysdimet,
                        predictor_columns = assumedpredictors,
                        assumed_targets = assumedtargets,
                        group_column = "SUBJECT_ID",
                        local_estimation = mebn.sampling,
                        local_model_cache = "models/BLMM_gamma",
                        stan_model_file = "mebn/BLMM_gamma.stan",
                        normalize_values = TRUE)
```

```
## [1] "fshdl"
## [1] "fsldl"
## [1] "fsins"
## [1] "fskol"
## [1] "fpgluk"
```

The visual comparison of the true and estimated distributions of blood concenrations shows that Gamma distribution follows the true distributions quite well.

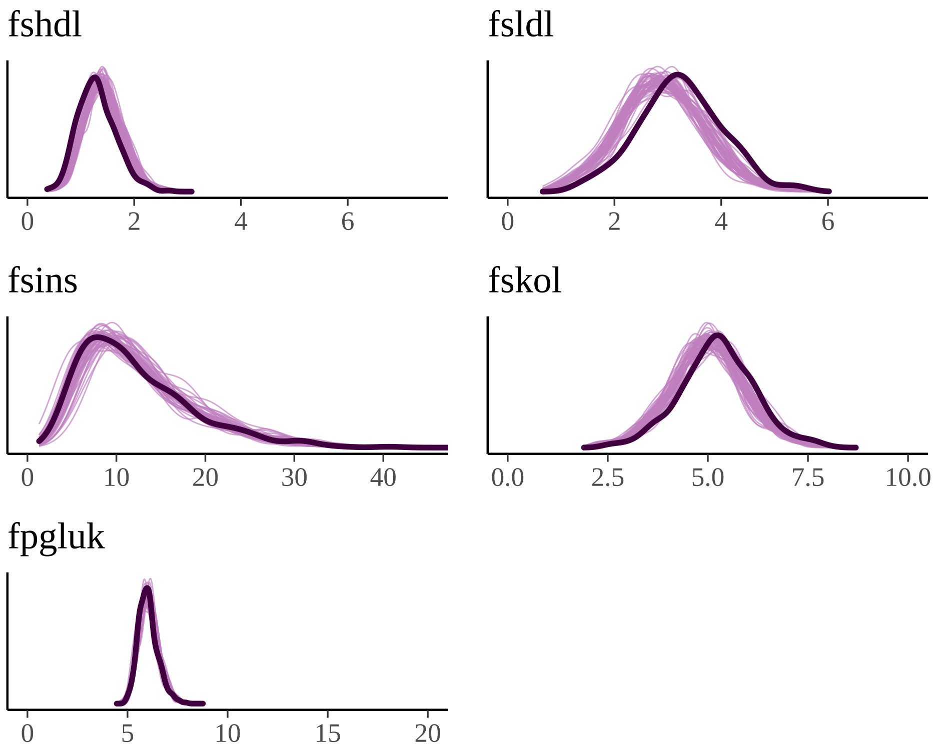

Supplementary Figure S 4: Visual posterior predictive check of local distributions with Gamma distribution. The figure is plotted with bayesplot package for R language (v 1.7.3, https://mc-stan.org/bayesplot).

The visual PPC inspection shows that the Gamma distribution fit generally better to responses than Normal distribution, but cholesterol concentrations are still systematically estimated too low (fsldl and fskol) or too high (fshdl).

Modeling correlated observations with an autocorrelation process

In the dataset the observations from subjects' food records and blood concentrations have one week response time. This might not be optimal time for all the responses and the successive observations might be correlated. For modeling the correlated observations, we add an autocorrelation process to the model by adding an estimated portion of previous measurement to the linear predictor

```
mu[n] += Y[n-1] * ar1
```

This coefficient 'ar1' is estimated separately for each response

```
## [1] "fshdl"
## [1] "fsldl"
## [1] "fsins"
## [1] "fskol"
## [1] "fpgluk"
```

The autocorrelation structure seems to decrease the variance of the model and correcting the systematical estimation errors from the previous model candidate

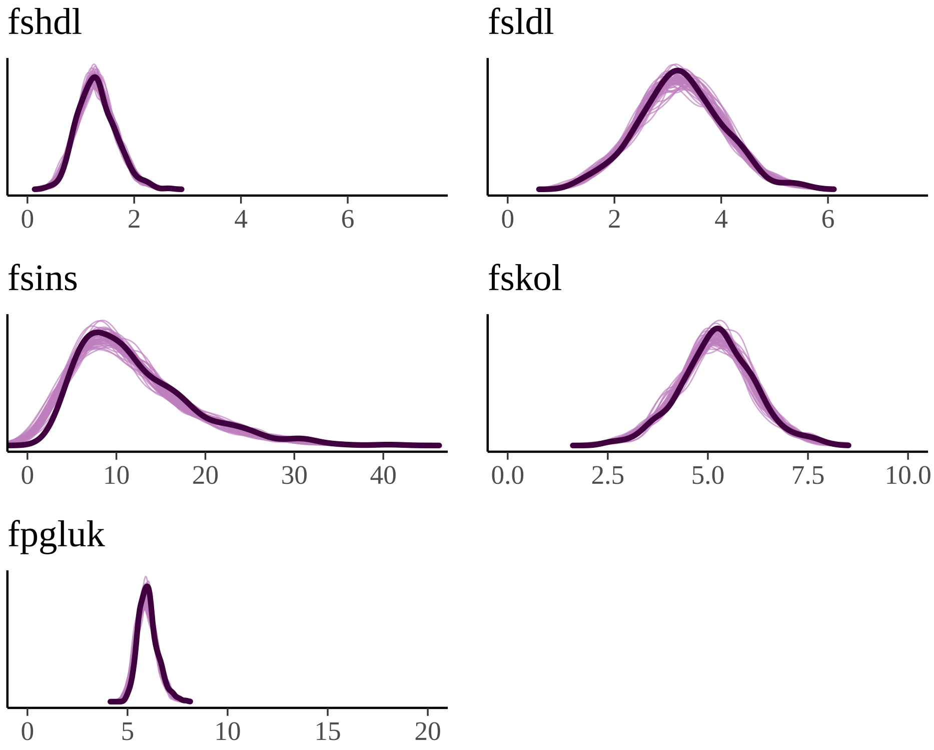

Supplementary Figure S 5: Visual posterior predictive check of local distributions with Gamma distribution and first-order autocorrelation process. The figure is plotted with bayesplot package for R language (v 1.7.3, https://mc-stan.org/bayesplot).

It is interesting also to compare the AR(1) coefficients of different local distributions at the graph. Blood insulin (fsins) has largest AR(1) coefficient indicating that successive measurements are most correlated and the values are not changing as rapidly as with other measurements. Credible interval for cholesterol measurements includes zero indicating that autocorrelation is very low or non-existent between two measurements.

Supplementary Table S 1: Comparison of autocorrelation coefficients for each distribution.

distribution	AR(1)	90%-CI lower	90%-CI upper
fshdl	0.00066	-0.00052	0.00179
fsldl	0.00064	-0.00180	0.00315
fsins	0.04553	0.02990	0.06127
fskol	0.00177	-0.00118	0.00472
fpgluk	0.00380	0.00157	0.00600

```
ar1_comparison <- mebn.AR_comparison(assumedtargets, "BLMM_gamma/ar1")
```

Skrinkage prior model

In our analysis, we use two models: 1) typical effects are better shown with previous model that has vague prior on beta coefficients, and 2) personal effects became clearer in a model that uses shrinkage prior on beta coefficients. This shrinkaged model is also faster to evaluate in k-fold setting and is possibly a better predictor.

The predictive ability of the model might be reduced if the model overfits to small nuances of the dataset. To overcome the possible overfitting, we apply a shrinkage prior "Regularized Horseshoe Shrinkage (RHS)" (Piironen and Vehtari 2017), also called Finnish horseshoe, on regression coefficients. It allows specifying a prior knowledge, or at least an educated guess, about the number of significant predictors for a target. Here we assume that one third of the nutrients might be relevant for any given blood concenration, and apply following parameters for the shrinkage

```
shrinkage_parameters <- within(list(),
{
    scale_icept  <- 1         # prior std for the intercept
    scale_global <- 0.02266   # scale for the half-t prior for tau:
                              # ((p0=7) / (D=22-7)) * (sigma = 1 / sqrt(n=106*4))
    nu_global    <- 1         # degrees of freedom for the half-t priors for tau
    nu_local     <- 1         # degrees of freedom for the half-t priors for lambdas
    slab_scale   <- 1         # slab scale for the regularized horseshoe
    slab_df      <- 1         # slab degrees of freedom for the regularized horseshoe
})
```

Next we fit the previous model with the Finnish horseshoe added. Besides the shrinkage parameters, we provide now the data in two sets: input data for estimation and target data to hold out for prediction.

```
# One RHS model with all data (no_holdout)
holdout_index <- rep(0, nrow(sysdimet))
```

```
initial_graph <- mebn.new_graph_with_randomvariables(datadesc)
sysdimet_gamma_ar1_rhs_pred <- mebn.bipartite_model(reaction_graph = initial_graph,
                                   inputdata = sysdimet,
                                   targetdata = holdout_index,
                                   predictor_columns = assumedpredictors,
                                   assumed_targets = assumedtargets,
                                   group_column = "SUBJECT_ID",
                                   local_estimation = mebn.sampling,
                                   local_model_cache = paste0("models/BLMM_gamma/ar1_rhs/no_holdout"),
```

```
                                stan_model_file = "mebn/BLMM_gamma_ar1_rhs_cv_ppc.stan",
                                reg_params = shrinkage_parameters,
                                normalize_values = TRUE)
```

```
## [1] "fshdl"
## [1] "fsldl"
## [1] "fsins"
## [1] "fskol"
## [1] "fpgluk"
```

```
write.graph(sysdimet_gamma_ar1_rhs_pred, "graphs/sysdimet_gamma_ar1_rhs.graphml", "graphml")
```

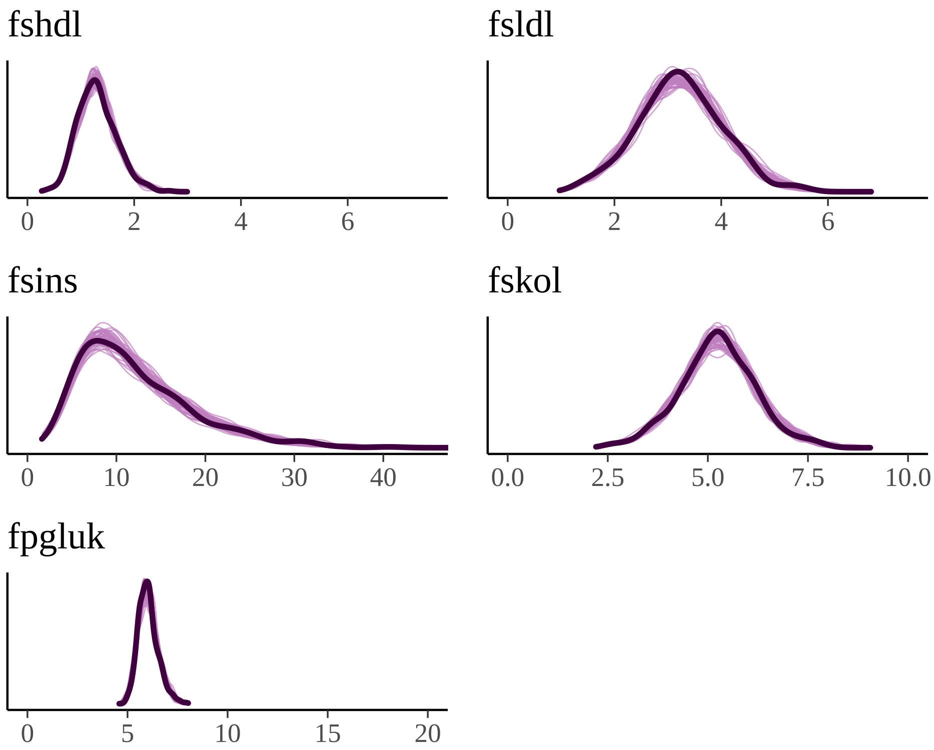

Supplementary Figure S 6: Visual posterior predictive check of local distributions with Gamma AR(1) distribution and regularized horseshoe prior on common effect coefficients. The figure is plotted with bayesplot package for R language (v 1.7.3, https://mc-stan.org/bayesplot).

Visual comparison with posterior predictive check seem to favor this model. Let us confirm the evaluation with numerical metrics.

Evaluation of the models' fit and predictive performance

Different model versions are compared with a normalized root mean squared error (NRMSE) and also with a classification test that allows comparison with other classification methods.

Comparison with in-sample data

Following compares different model candidates by using normalized root mean squared error (NRMSE) metrics. Normalization allows comparing the prediction accuracy of responses with different scales.

```
## [1] "fsins"
```

```
initial_graph <- mebn.new_graph_with_randomvariables(datadesc)
only_fsins <- assumedtargets[assumedtargets$Name=="fsins",]

fsins_normal_ar1_rhs <- mebn.bipartite_model(reaction_graph = initial_graph,
                                    inputdata = sysdimet,
                                    targetdata = holdout_index,
                                    predictor_columns = assumedpredictors,
                                    assumed_targets = only_fsins,
                                    group_column = "SUBJECT_ID",
                                    local_estimation = mebn.sampling,
                                    local_model_cache = paste0("models/BLMM_normal/ar1_rhs/no_holdout"),
                                    stan_model_file = "mebn/BLMM_normal_ar1_rhs_cv_ppc.stan",
                                    reg_params = shrinkage_parameters,
                                    normalize_values = TRUE)
```

```
## [1] "fsins"
```

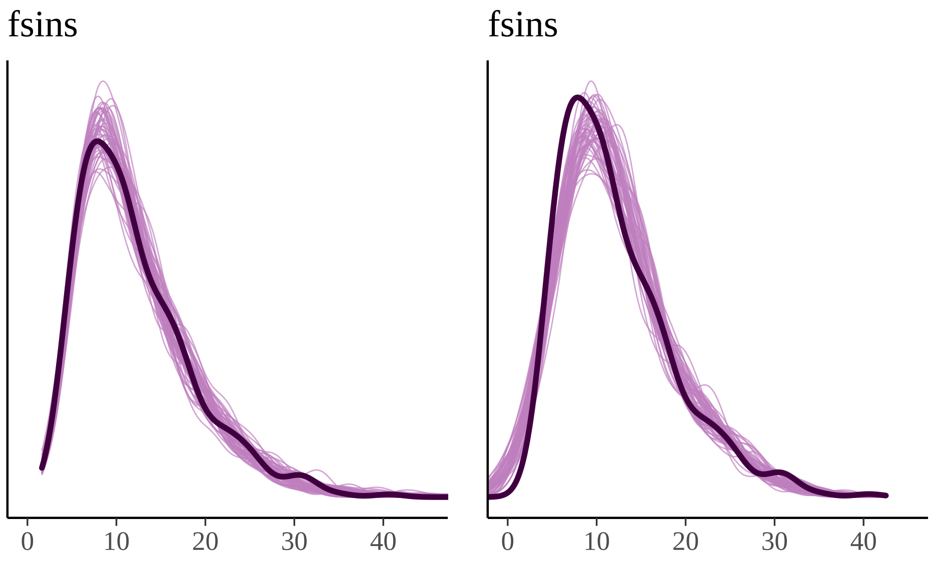

Supplementary Figure S 7: Plot on the left is a PPC of Gamma distributed fsins-response and the right plot is a normally distributed response that fits better with lower mean squared error. The figure is plotted with bayesplot package for R language (v 1.7.3, https://mc-stan.org/bayesplot).

Next, we contruct a EXPFAM AR(1) model that has this normally distributed fsins-response and otherwise Gamma distributed blood concentration responses.

```
## [1] "fshdl"
## [1] "models/BLMM_gamma/ar1"
```

Supplementary Table S 2: Model candidates were compared with a normalized root mean squared error (NRMSE) separately for each concentration and with mean accuracy of whole Bayesian network. The last final versions (Gamma AR(1) RHS CV and Expfam AR(1) RHS CV) were evaluated with the cross-validation.

	NRMSE					
**Model**	**HDL-chol.**	**LDL-chol.**	**Insulin**	**Total chol.**	**Glucose**	**BN Mean**
Normal	0.04	0.06	0.22	0.06	0.06	0.09
Gamma	0.02	0.06	0.28	0.05	0.03	0.09
Gamma AR(1)	0.01	0.03	0.27	0.04	0.03	0.07
Gamma AR(1) RHS	0.01	0.03	0.29	0.04	0.03	0.08
EXPFAM AR(1)	0.01	0.03	0.23	0.04	0.03	0.07
EXPFAM AR(1) RHS	0.01	0.03	0.23	0.04	0.03	0.07
Gamma AR(1) RHS CV	0.02	0.05	0.44	0.06	0.05	0.12
EXPFAM AR(1) RHS CV	0.02	0.05	0.45	0.06	0.05	0.13

```
## [1] "fsldl"
## [1] "models/BLMM_gamma/ar1"
## [1] "fsins"
## [1] "models/BLMM_normal/ar1"
## [1] "fskol"
## [1] "models/BLMM_gamma/ar1"
## [1] "fpgluk"
## [1] "models/BLMM_gamma/ar1"
```

```
## [1] "fshdl"
## [1] "models/BLMM_gamma/ar1_rhs/no_holdout"
## [1] "fsldl"
## [1] "models/BLMM_gamma/ar1_rhs/no_holdout"
## [1] "fsins"
## [1] "models/BLMM_normal/ar1_rhs/no_holdout"
## [1] "fskol"
## [1] "models/BLMM_gamma/ar1_rhs/no_holdout"
## [1] "fpgluk"
## [1] "models/BLMM_gamma/ar1_rhs/no_holdout"
```

Predictive accuracy cross-validated MEBN

Execution of the k-fold cross-validation is done in a separated notebook (cross-validation.Rmd) as it takes substantial amount of time to run. The results are stored and load for analysis here. For the cross-validation we partitioned the observations in data to 12 partitions for weeks 4,8 and 12. Then each of the data partitions in turn is left out from model estimation. This allows us to make predictions for unseen data.

Evaluation with classification metrics

Normalized root mean squared error (NRMSE) is good for comparing model candidates, but it does not say much about the actual usefulness of the model. For personal nutrition guiding, it is important to predict how given diet will affect the future blood concentration values. In the standard NRMSE comparison, the error between true and predicted values were compared, but a custom classification test can be also used. It tells if the given diet raises or lowers the future blood concentrations compared to past measurement.

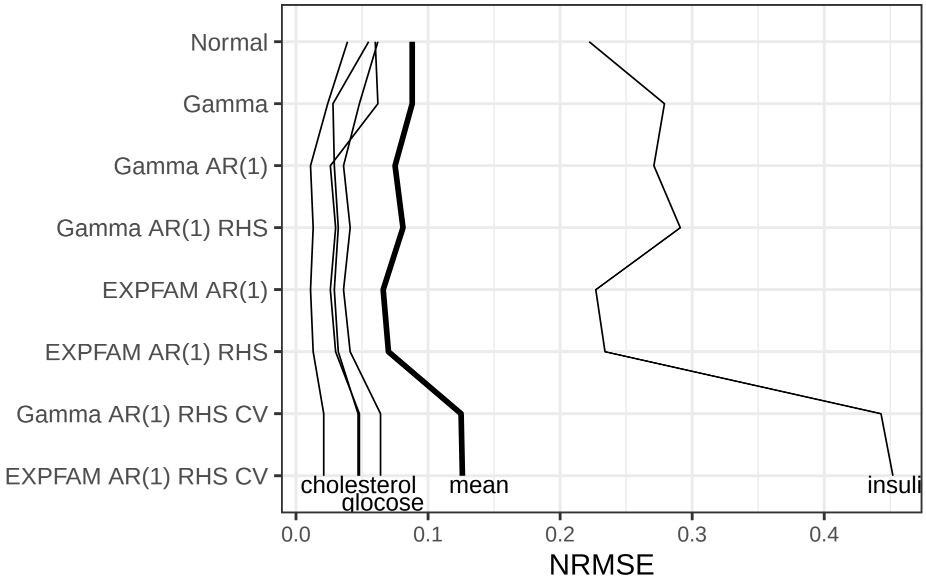

Supplementary Figure S 8: Comparison of NRMSE metrics of different model versions. Normaized error is measured each local concentration distribution and for the whole network as mean of local distribution errors. The figure is plotted with ggplot2 package for R language (v 3.3.2, https://ggplot2.tidyverse.org).

Supplementary Table S 3: Comparison of modeling methods in a classification test where the direction of change for future concentration is predicted based on its past value and past diet.

	Prediction accuracy for the direction of change				
**Method**	**HDL-chol.**	**LDL-chol.**	**Insulin**	**Glucose**	**Total chol.**
MEBN	81%	85%	85%	74%	83%
MEBN RHS	78%	81%	84%	70%	80%
MEBN RHS CV	63%	67%	68%	59%	64%
XGBoost CV	61%	69%	68%	60%	71%

Classification test allows also to compare MEBN with other classification methods. XGBoost is a well-performing decision tree method. It is a non-parametric machine learning method that learns personal reactions only by example. Following compares the cross-validated predictive accuracy of XGBoost with MEBN predictions (MEBN CV) that uses EXPFAM AR(1) RHS-model. Also in-sample MEBN fit with non-shrinked EXPFAM AR(1) is given for comparison. This best predicting model is used for dataset analysis at rest of this notebook.

Summary of the model comparison

This table summarizes how accurately we can predict if blood concenration increase or decrease when the current level and a diet are known.

Typical effects of nutrition and personal variance

We have now reached a reasonably good fit for the local distributions of the Bayesian network. Our code has extracted a graphical model from these posterior distributions of random variables and parameters. The

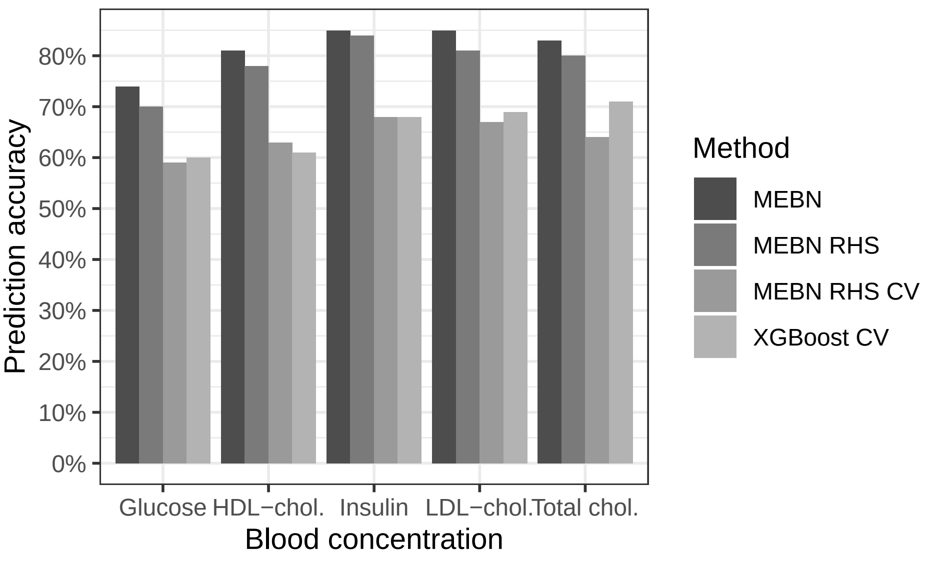

Supplementary Figure S 9: Different modeling methods were compared in a classification test where the direction of change for future concentration is predicted based on its past value and past diet. The proposed mixed-effect Bayesian network (MEBN) is compared with Extreme Gradient Boosting (XGBoost) decision tree method. The best-fitting EXPFAM AR(1) model version is used in this comparison with and without shrinkage prior (RHS). Cross-validated (CV) prediction accuracy is similar for both methods. The in-sample accuracy of MEBN is substantially higher, and these estimates are used in drawing conclusions at the clustering and personal models in this article. The figure is plotted with ggplot2 package for R language (v 3.3.2, https://ggplot2.tidyverse.org).

graph consists mean values of the posteriors and also their credible intervals. This is a lighter data structure for further analysis and allows also graph operations besides the regression modeling.

Besides the random variables, the graph also includes the regression coefficients β and b that denote the typical and personal magnitudes of the effects for different nutrients. For better overview, let us plot the graph of typical nutritional effects.

We can examine closer the effects in the previous graph. In following, we show 90% credible interval for each effect and sort them by variance between persons. These effects with high variance between persons are interesting to us.

These are the effect with most difference between subjects. Let us the examine closer those of the effects that have most probable variance over 0.30 and plot them as a graph

Sensitivity analysis

In Table S4, we provide a sensitivity analysis on how the graph estimates with either vague or shrinkage priors on beta parameters. It can be seen that standard deviations of personal effects are not sensitive to prior choice. In the actual personal effects, the shrinkage prior puts more weight on personal variations and less weight on general part of the effect.

In addition, the effect parameters with most personal variation are provided in Table S5

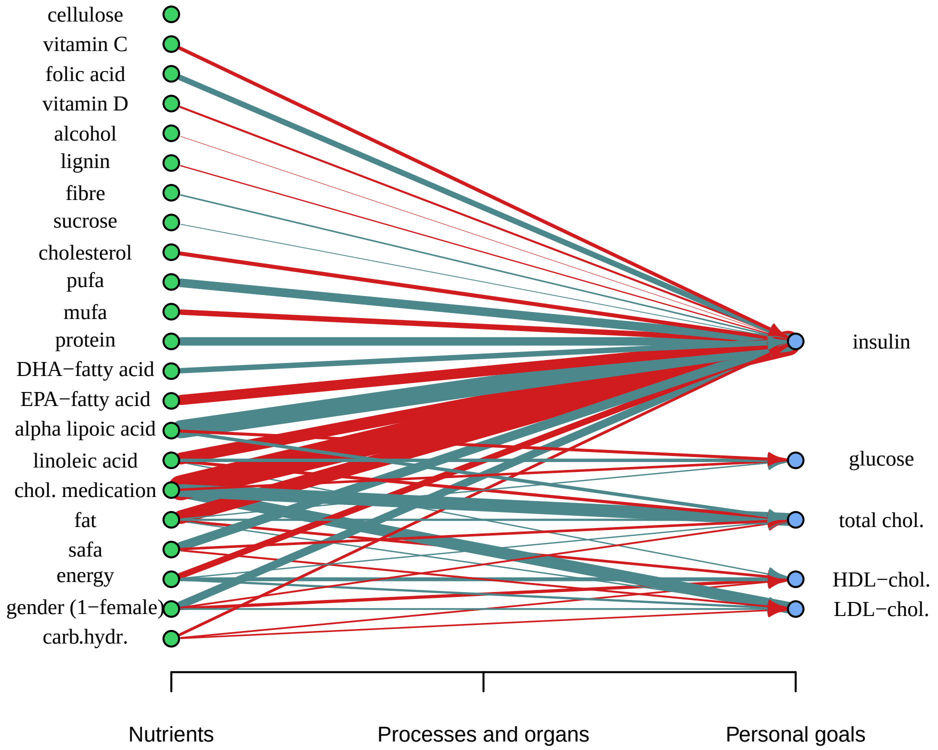

Supplementary Figure S 10: This visualization shows mean of posterior for typical coefficients as weight of the connection between blood concentrations and nutrients at diet affecting it. For clarity, the visualization only shows 15 principal components explaining the variance of each blood concentrations. The figure is plotted with iGraph package for R language (v 1.2.6, https://igraph.org/r).

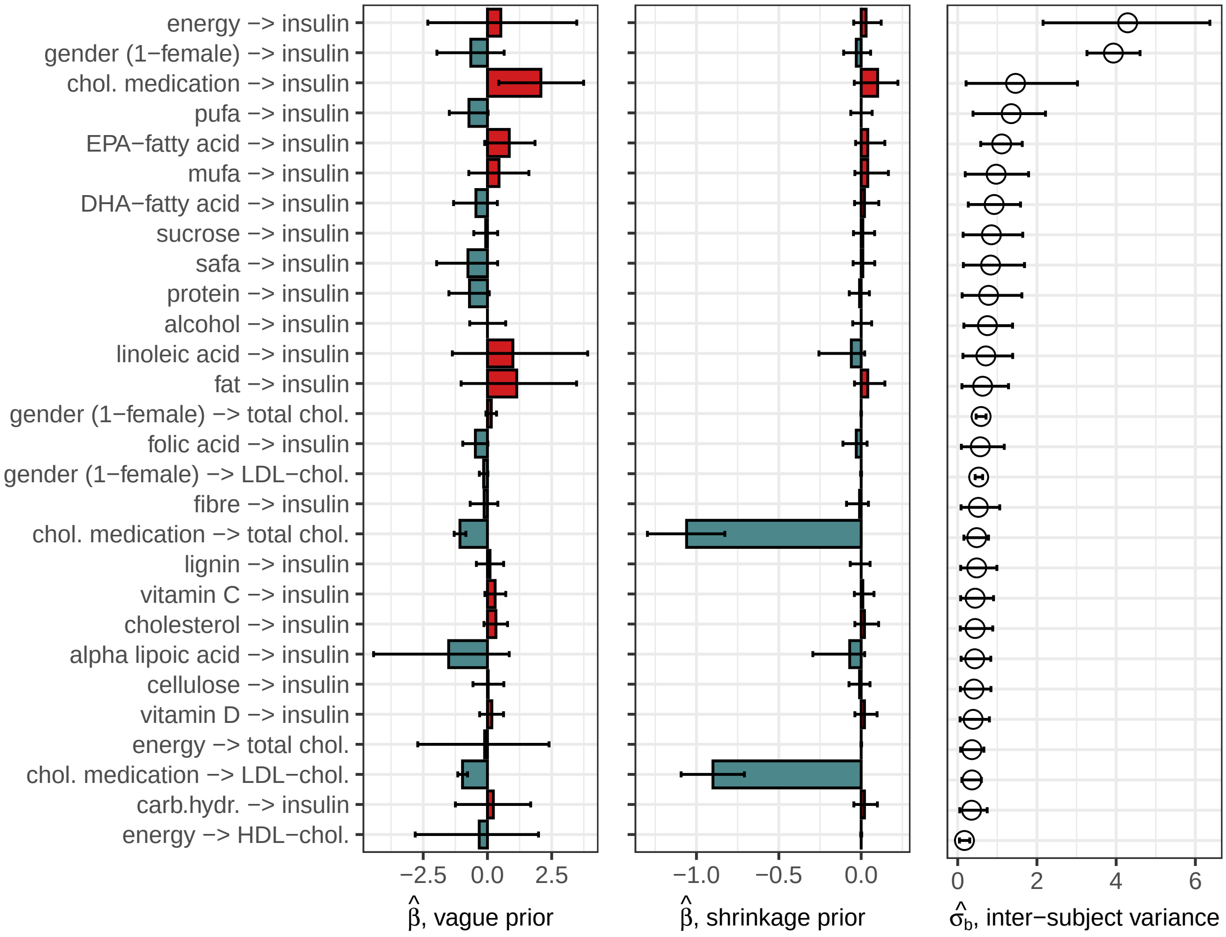

Supplementary Figure S 11: List of nutritional effects where the effect is either generally strong or it has high variation between subjects. The figure shows posterior means with credible intervals and compares the effect of shrinkage prior to vague prior. The figure is plotted with ggplot2 package for R language (v 3.3.2, https://ggplot2.tidyverse.org).

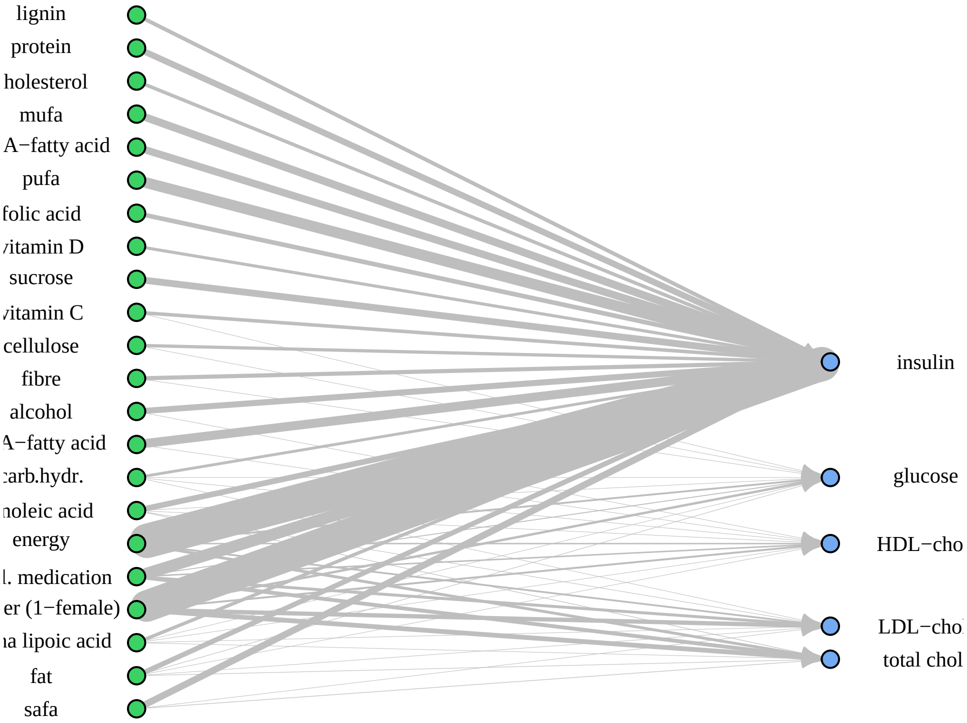

Supplementary Figure S 12: Graph visualization of inter-subject variation in nutritional effects. The effect of energy intake has one of the largest inter-subject variances at insulin response. The figure is plotted with iGraph package for R language (v 1.2.6, https://igraph.org/r).

Supplementary Table S 4: Sensitivity analysis of prior choice.

		Inter-subject variation ($\hat{\sigma_b}$)		Personal effect ($\hat{\beta} + \hat{\mathbf{b}}$)			
				Vague prior		RHS prior	
**Nutrient or feature**	**Concentration**	**Vague prior**	**RHS prior**	**Min.**	**Max.**	**Min.**	**Max.**
energy	insulin	4.29 [2.16; 6.37]	4.83	0.12 [-4; 4.43]	1.12 [-3; 6.09]	-0.11 [-2.41; 1.03]	0.35 [-0.65; 3.43]
gender (1-female)	insulin	3.93 [3.26; 4.6]	4.02	-1.03 [-4.82; 1.64]	0.19 [-2.59; 5.56]	-0.33 [-4.52; 1.62]	0.49 [-1.62; 5.94]
chol. medication	insulin	1.46 [0.21; 3.02]	1.52	-1.29 [-7.5; 3.7]	8.63 [2.18; 15.94]	-2.8 [-8.85; 2.33]	8.59 [1.15; 16.27]
pufa	insulin	1.35 [0.39; 2.22]	1.28	-1.12 [-3.47; 0.29]	-0.4 [-1.81; 1.59]	-0.2 [-2.35; 0.66]	0.2 [-0.61; 2.1]
EPA-fatty acid	insulin	1.11 [0.58; 1.63]	1.08	0.23 [-1.06; 1.61]	1.72 [-0.14; 4.8]	-0.34 [-2.73; 0.86]	1.1 [-0.18; 3.5]
mufa	insulin	0.97 [0.19; 1.79]	0.89	-0.96 [-3.83; 1.29]	2.27 [-0.51; 6.01]	-1.13 [-3.47; 0.45]	1.73 [-0.21; 5]
DHA-fatty acid	insulin	0.92 [0.27; 1.59]	0.96	-0.62 [-2.28; 0.72]	-0.1 [-1.47; 1.9]	-0.08 [-1.6; 0.69]	0.34 [-0.33; 2.36]
sucrose	insulin	0.85 [0.14; 1.64]	0.83	-0.28 [-1.83; 0.63]	0.12 [-0.75; 1.82]	-0.07 [-1.25; 0.51]	0.12 [-0.45; 1.71]
safa	insulin	0.83 [0.14; 1.69]	0.77	-1.46 [-4.22; 0.48]	0.3 [-2.09; 4.13]	-0.43 [-3.11; 0.57]	0.85 [-0.4; 4.7]
protein	insulin	0.78 [0.11; 1.62]	0.74	-0.97 [-2.93; 0.42]	-0.4 [-1.88; 2.06]	-0.14 [-1.81; 0.5]	0.12 [-0.75; 2.09]
alcohol	insulin	0.75 [0.16; 1.39]	0.71	-0.17 [-1.49; 0.89]	0.11 [-0.98; 1.48]	-0.08 [-1.13; 0.4]	0.04 [-0.57; 1.02]
linoleic acid	insulin	0.71 [0.13; 1.39]	0.65	0.18 [-2.84; 4.95]	0.91 [-1.81; 5.68]	-0.28 [-1.98; 0.24]	0.04 [-0.55; 1.14]
fat	insulin	0.63 [0.11; 1.28]	0.54	0.7 [-2.75; 4.28]	1.73 [-1.37; 6.26]	-0.1 [-2.13; 1.01]	0.46 [-0.58; 3.59]
gender (1-female)	total chol.	0.59 [0.47; 0.72]	0.50	-0.7 [-1.98; 0.24]	0.82 [0.01; 1.94]	-0.5 [-1.61; 0.09]	0.48 [-0.1; 1.57]
folic acid	insulin	0.57 [0.09; 1.18]	0.55	-0.66 [-2.22; 0.27]	-0.22 [-1.15; 1.6]	-0.13 [-1.48; 0.43]	0.12 [-0.52; 1.67]
gender (1-female)	LDL-chol.	0.53 [0.44; 0.63]	0.48	-0.67 [-1.63; -0.02]	0.36 [-0.27; 1.36]	-0.31 [-1.32; 0.1]	0.27 [-0.12; 1.16]
fibre	insulin	0.52 [0.08; 1.06]	0.45	-0.24 [-1.37; 0.7]	0 [-0.91; 1.23]	-0.05 [-1.08; 0.59]	0.05 [-0.49; 1.07]
chol. medication	total chol.	0.48 [0.16; 0.78]	0.41	-1.39 [-2.5; -0.83]	-0.71 [-1.32; 0.5]	-1.48 [-2.62; -0.86]	-0.75 [-1.35; 0.44]
lignin	insulin	0.48 [0.08; 0.99]	0.48	-0.4 [-2.75; 0.85]	0.68 [-0.43; 2.48]	-0.49 [-2.88; 0.47]	0.42 [-0.31; 2.16]
vitamin C	insulin	0.44 [0.07; 0.9]	0.42	-0.76 [-2.67; 0.65]	2.43 [0.43; 4.82]	-0.98 [-2.75; 0.35]	2.4 [0.23; 4.77]
cholesterol	insulin	0.44 [0.06; 0.88]	0.39	0.27 [-0.75; 1.13]	0.39 [-0.47; 1.48]	-0.02 [-0.81; 0.56]	0.04 [-0.44; 0.85]
alpha lipoic acid	insulin	0.43 [0.09; 0.83]	0.38	-1.69 [-6.65; 1.19]	-0.86 [-5.63; 1.98]	-0.31 [-2.13; 0.24]	0.05 [-0.54; 1.04]
cellulose	insulin	0.41 [0.07; 0.84]	0.38	-0.12 [-1.76; 0.96]	0.23 [-0.94; 1.86]	-0.1 [-1.5; 0.59]	0.11 [-0.48; 1.58]
vitamin D	insulin	0.39 [0.06; 0.8]	0.37	-0.04 [-2.16; 1.02]	0.52 [-0.32; 1.97]	-0.12 [-1.88; 0.67]	0.28 [-0.26; 1.73]
chol. medication	LDL-chol.	0.36 [0.11; 0.6]	0.29	-1.08 [-1.77; -0.7]	-0.81 [-1.23; 0.12]	-1.01 [-1.69; -0.62]	-0.79 [-1.18; 0.08]
energy	total chol.	0.36 [0.07; 0.66]	0.38	-0.18 [-3.76; 3.27]	-0.07 [-3.67; 3.36]	-0.01 [-0.18; 0.06]	0.01 [-0.08; 0.21]
carb.hydr.	insulin	0.35 [0.05; 0.74]	0.33	-0.29 [-3.11; 1.96]	1 [-1.44; 4.29]	-0.34 [-2.57; 0.58]	0.58 [-0.46; 3.31]
gender (1-female)	glucose	0.3 [0.24; 0.36]	0.21	-0.1 [-0.48; 0.12]	0 [-0.22; 0.38]	-0.13 [-0.66; 0.07]	0.1 [-0.1; 0.6]
gender (1-female)	HDL-chol.	0.24 [0.2; 0.29]	0.20	0.09 [-0.29; 0.34]	0.53 [0.21; 0.96]	-0.02 [-0.41; 0.31]	0.6 [0.19; 1.05]
energy	glucose	0.23 [0.05; 0.41]	0.19	-0.06 [-3.32; 3.2]	0.06 [-3.17; 3.35]	-0.01 [-0.1; 0.05]	0.01 [-0.04; 0.13]
energy	LDL-chol.	0.23 [0.04; 0.47]	0.23	-0.2 [-3.53; 3.03]	-0.12 [-3.45; 3.1]	-0.01 [-0.14; 0.06]	0.01 [-0.06; 0.17]
chol. medication	HDL-chol.	0.18 [0.07; 0.29]	0.21	-0.19 [-0.54; 0.02]	0.08 [-0.17; 0.49]	-0.24 [-0.63; 0.03]	0.17 [-0.08; 0.64]
energy	HDL-chol.	0.17 [0.04; 0.3]	0.21	-0.29 [-3.63; 2.77]	-0.24 [-3.6; 2.77]	0 [-0.06; 0.03]	0.01 [-0.02; 0.07]
cellulose	LDL-chol.	0.14 [0.06; 0.21]	0.07	-0.03 [-0.17; 0.08]	0 [-0.11; 0.15]	0 [-0.09; 0.06]	0 [-0.05; 0.09]
cellulose	total chol.	0.13 [0.03; 0.22]	0.05	-0.02 [-0.17; 0.12]	0 [-0.13; 0.16]	0 [-0.09; 0.06]	0 [-0.06; 0.11]
sucrose	glucose	0.12 [0.04; 0.2]	0.04	-0.06 [-0.27; 0.05]	-0.01 [-0.11; 0.17]	0 [-0.09; 0.05]	0.01 [-0.04; 0.13]
chol. medication	glucose	0.12 [0.02; 0.24]	0.17	0.04 [-0.52; 0.36]	0.39 [0.06; 0.96]	-0.05 [-0.48; 0.16]	0.14 [-0.09; 0.73]
sucrose	total chol.	0.12 [0.02; 0.22]	0.05	-0.01 [-0.29; 0.13]	0.06 [-0.05; 0.22]	-0.01 [-0.23; 0.08]	0.04 [-0.03; 0.27]

Supplementary Table S 5: These 38 (of all 110) effects from nutrients and other modeled features to blood concentrations had the highest variations between the studied subjects. The estimated standard deviation of personal effects varies from 4.29 to 0.02, and this table is sorted to include the most varying effects with the standard deviation over 0.10.

			Personal effect ($\hat{\beta} + \hat{\mathbf{b}}$)	
**Nutrient or feature**	**Concentration**	**Inter-subject variation ($\hat{\sigma_b}$)**	**Min.**	**Max.**
energy	insulin	4.29 [2.16; 6.37]	0.12 [-4; 4.43]	1.12 [-3; 6.09]
gender (1-female)	insulin	3.93 [3.26; 4.6]	-1.03 [-4.82; 1.64]	0.19 [-2.59; 5.56]
chol. medication	insulin	1.46 [0.21; 3.02]	-1.29 [-7.5; 3.7]	8.63 [2.18; 15.94]
pufa	insulin	1.35 [0.39; 2.22]	-1.12 [-3.47; 0.29]	-0.4 [-1.81; 1.59]
EPA-fatty acid	insulin	1.11 [0.58; 1.63]	0.23 [-1.06; 1.61]	1.72 [-0.14; 4.8]
mufa	insulin	0.97 [0.19; 1.79]	-0.96 [-3.83; 1.29]	2.27 [-0.51; 6.01]
DHA-fatty acid	insulin	0.92 [0.27; 1.59]	-0.62 [-2.28; 0.72]	-0.1 [-1.47; 1.9]
sucrose	insulin	0.85 [0.14; 1.64]	-0.28 [-1.83; 0.63]	0.12 [-0.75; 1.82]
safa	insulin	0.83 [0.14; 1.69]	-1.46 [-4.22; 0.48]	0.3 [-2.09; 4.13]
protein	insulin	0.78 [0.11; 1.62]	-0.97 [-2.93; 0.42]	-0.4 [-1.88; 2.06]
alcohol	insulin	0.75 [0.16; 1.39]	-0.17 [-1.49; 0.89]	0.11 [-0.98; 1.48]
linoleic acid	insulin	0.71 [0.13; 1.39]	0.18 [-2.84; 4.95]	0.91 [-1.81; 5.68]
fat	insulin	0.63 [0.11; 1.28]	0.7 [-2.75; 4.28]	1.73 [-1.37; 6.26]
gender (1-female)	total chol.	0.59 [0.47; 0.72]	-0.7 [-1.98; 0.24]	0.82 [0.01; 1.94]
folic acid	insulin	0.57 [0.09; 1.18]	-0.66 [-2.22; 0.27]	-0.22 [-1.15; 1.6]
gender (1-female)	LDL-chol.	0.53 [0.44; 0.63]	-0.67 [-1.63; -0.02]	0.36 [-0.27; 1.36]
fibre	insulin	0.52 [0.08; 1.06]	-0.24 [-1.37; 0.7]	0 [-0.91; 1.23]
chol. medication	total chol.	0.48 [0.16; 0.78]	-1.39 [-2.5; -0.83]	-0.71 [-1.32; 0.5]
lignin	insulin	0.48 [0.08; 0.99]	-0.4 [-2.75; 0.85]	0.68 [-0.43; 2.48]
vitamin C	insulin	0.44 [0.07; 0.9]	-0.76 [-2.67; 0.65]	2.43 [0.43; 4.82]
cholesterol	insulin	0.44 [0.06; 0.88]	0.27 [-0.75; 1.13]	0.39 [-0.47; 1.48]
alpha lipoic acid	insulin	0.43 [0.09; 0.83]	-1.69 [-6.65; 1.19]	-0.86 [-5.63; 1.98]
cellulose	insulin	0.41 [0.07; 0.84]	-0.12 [-1.76; 0.96]	0.23 [-0.94; 1.86]
vitamin D	insulin	0.39 [0.06; 0.8]	-0.04 [-2.16; 1.02]	0.52 [-0.32; 1.97]
chol. medication	LDL-chol.	0.36 [0.11; 0.6]	-1.08 [-1.77; -0.7]	-0.81 [-1.23; 0.12]
energy	total chol.	0.36 [0.07; 0.66]	-0.18 [-3.76; 3.27]	-0.07 [-3.67; 3.36]
carb.hydr.	insulin	0.35 [0.05; 0.74]	-0.29 [-3.11; 1.96]	1 [-1.44; 4.29]
gender (1-female)	glucose	0.3 [0.24; 0.36]	-0.1 [-0.48; 0.12]	0 [-0.22; 0.38]
gender (1-female)	HDL-chol.	0.24 [0.2; 0.29]	0.09 [-0.29; 0.34]	0.53 [0.21; 0.96]
energy	glucose	0.23 [0.05; 0.41]	-0.06 [-3.32; 3.2]	0.06 [-3.17; 3.35]
energy	LDL-chol.	0.23 [0.04; 0.47]	-0.2 [-3.53; 3.03]	-0.12 [-3.45; 3.1]
chol. medication	HDL-chol.	0.18 [0.07; 0.29]	-0.19 [-0.54; 0.02]	0.08 [-0.17; 0.49]
energy	HDL-chol.	0.17 [0.04; 0.3]	-0.29 [-3.63; 2.77]	-0.24 [-3.6; 2.77]
cellulose	LDL-chol.	0.14 [0.06; 0.21]	-0.03 [-0.17; 0.08]	0 [-0.11; 0.15]
cellulose	total chol.	0.13 [0.03; 0.22]	-0.02 [-0.17; 0.12]	0 [-0.13; 0.16]
sucrose	glucose	0.12 [0.04; 0.2]	-0.06 [-0.27; 0.05]	-0.01 [-0.11; 0.17]
chol. medication	glucose	0.12 [0.02; 0.24]	0.04 [-0.52; 0.36]	0.39 [0.06; 0.96]
sucrose	total chol.	0.12 [0.02; 0.22]	-0.01 [-0.29; 0.13]	0.06 [-0.05; 0.22]

Supplementary Table S 6: Number of patients using the cholesterol-lowering medication in the found clusters.

	1	2	3	4
No medication	288	0	20	0
Chol. med.	44	44	0	28

Finding the clusters of personal reaction types

Although there are personal differences, it is likely that everyone is not behaving uniquely but there might exist similar groups of behavior. We can analyze personal differences in two ways. We can look the absolute magnitudes of effects and we can also look how persons differ from typical behavior or mean of the effect.

We can now take the personal estimations of the effects and see, if they form clusters

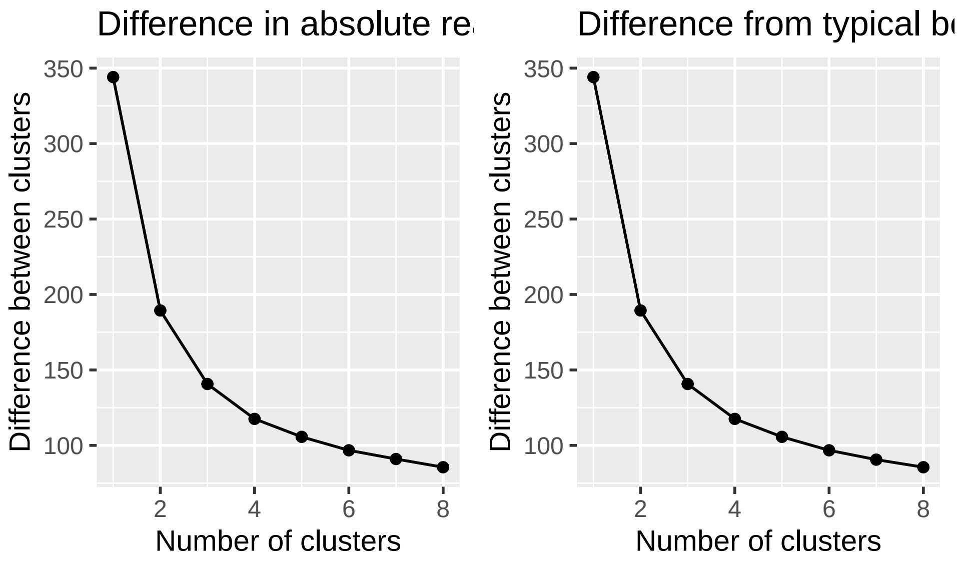

Supplementary Figure S 13: The elbow plot of k-means clustering shows that difference between clusters decreases when more than four clusters are considered. These clusters include all the predictors, nutrients and the cholesterol lowering medication. The figure is plotted with ggplot2 package for R language (v 3.3.2, https://ggplot2.tidyverse.org).

By looking the previous elbow-plot diagram we see that there are four clearly identifiable groups between subjects.

The effect of cholesterol medication

At this plot, zero of X-axis denotes a typical behavior of that particular reaction, and bars denote a deviation from this typical mean.

Cholesterol medication seems to dominate the clusters. There are subjects for who cholesterol medication raises the blood insulin levels more than on average and for other group the raise is less than on average.

By looking at the absolute effect of the cholesterol medication it seems that there separates two groups where one it is a significant factor in explaining blood insulin level, and other group where it does not play virtually any role. But does these subjects even take cholesterol medications? Let's create a cross tabulation of observations to explore this effect more closely.

This indicates that subjects in clusters 2 and 4 are indeed taking cholesterol medication.

The average insulin concentrations in these clusters are

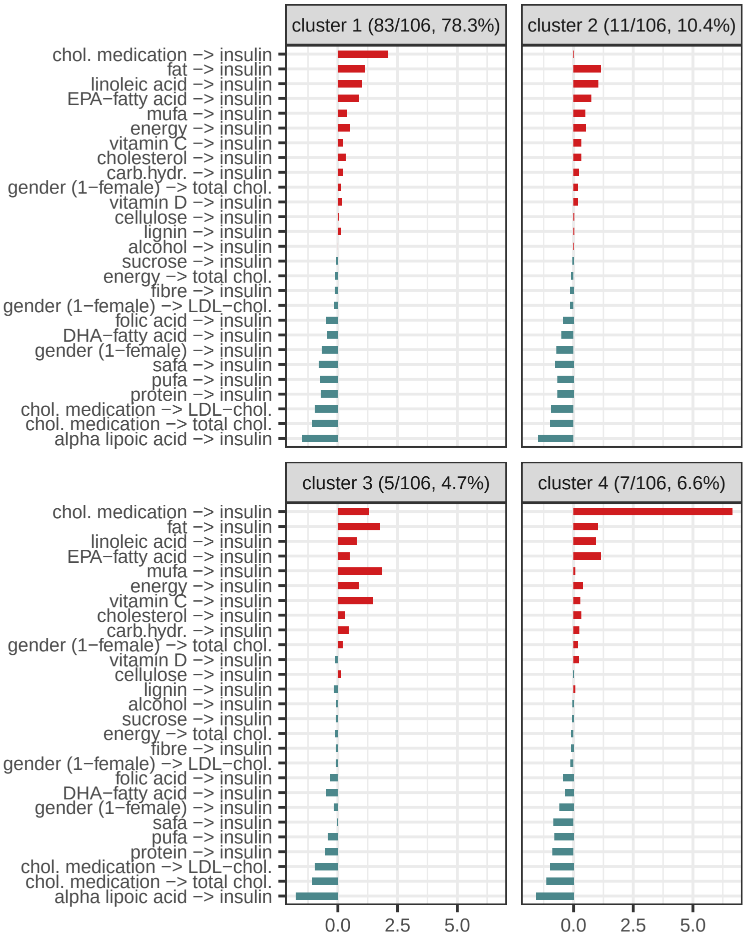

Supplementary Figure S 14: Four most clearly separating clusters of personal reactions when all the predictors are considered, including cholesterol medication. The clustering is done for posterior means of personal effects. The effect of cholesterol-lowering medication to the insulin concentration dominates the personally varying effects. Besides decreasing the cholesterol concentration, the medication has a side effect of increasing insulin concentration for most of the subjects. Yet, there exists a large variation. The increasing effect is dramatic for 6,6% of the subjects in cluster 4, but for 10,4% of subjects in cluster 2 the effect is non-existing. The figure is plotted with ggplot2 package for R language (v 3.3.2, https://ggplot2.tidyverse.org).

Supplementary Table S 7: Average insulin concentrations (mU/l) within the found clusters.

**cluster**	**average blood insulin**
1	10.95
2	8.07
3	25.48
4	23.11

There are 9 (=36/4) and 7 (=28/4) subjects in clusters 2 and 4 who are taking cholesterol medication. For subjects in the cluster 4 the average insulin level is the medication seems to increase the insulin concentration quite much, but for subjects in the cluster 2 not much at all.

Clustering with nutritional effects only

Both gender and cholesterol medication are unchanging factors at the dataset. Let us next remove those from clustering features and see how the clusters form based on nutritional effects only.

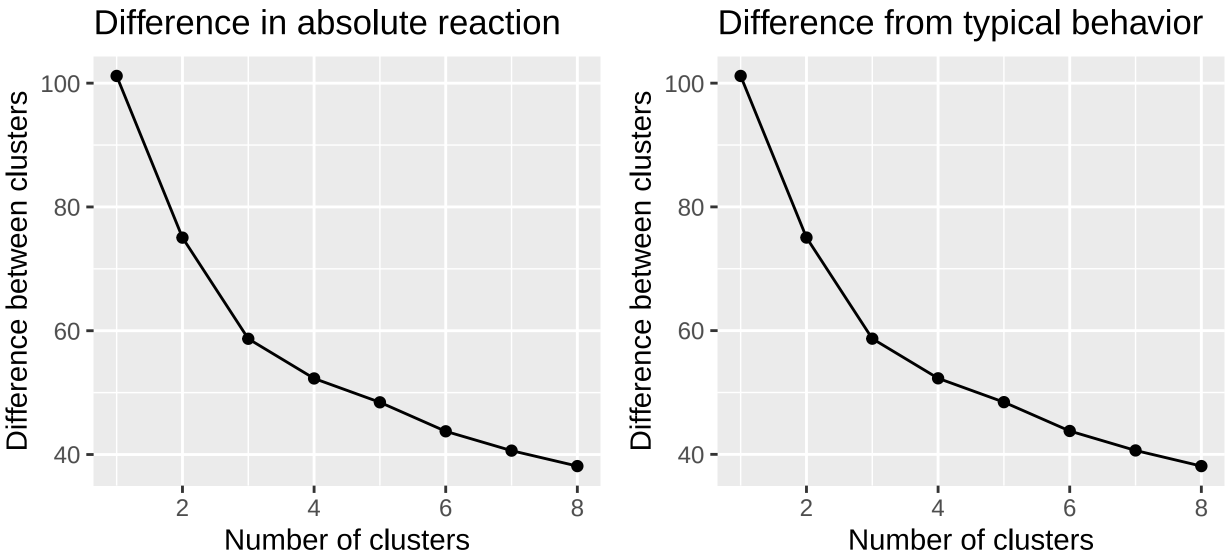

Supplementary Figure S 15: Four clusters separated also when only the nutrient predictors of concentrations were considered. The figure is plotted with ggplot2 package for R language (v 3.3.2, https://ggplot2.tidyverse.org).

In clusters 1 and 2 there are subjects whose insulin concentrations decrease and increase more than on average.

Personally predicted reaction types

During the cross-validation we estimated personal models for each subject. Next we illustrate the differences within personal reaction types by picking few most distant ones.

We can then compare the visualizations of the personal reaction graphs. The effect of cholesterol medication is most significant difference, but other differences exist as well.

Following table compares some of the distinguishing differences between these subjects

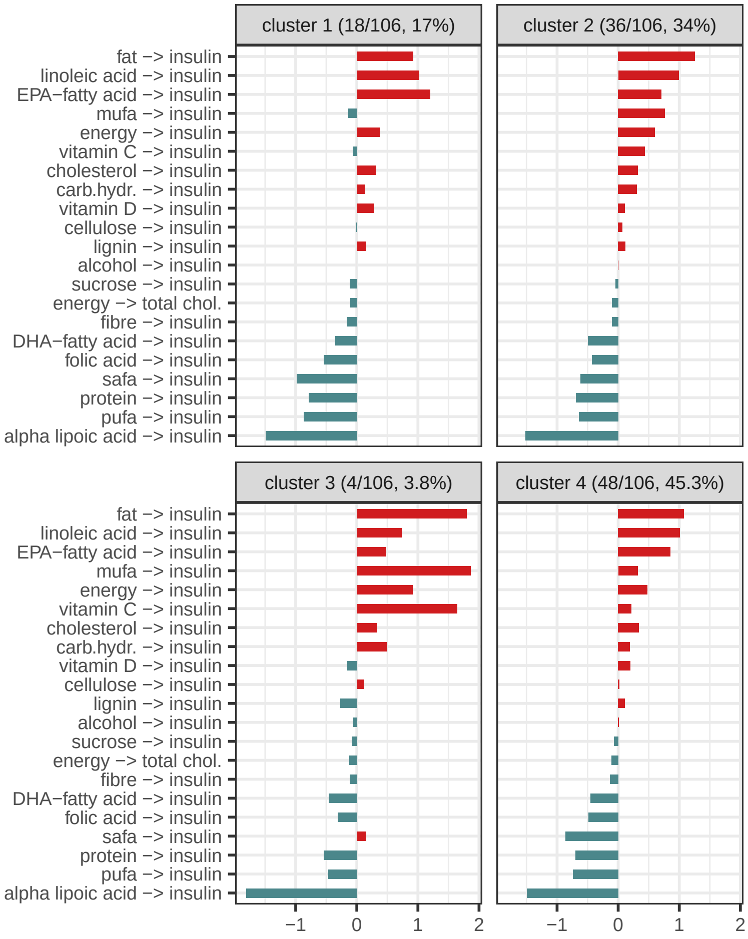

Supplementary Figure S 16: Four clusters separated also when only the effects of nutrients were considered, and the cholesterol medication was left out. The modeled Bayesian network includes 110 different effects of nutrition, but these figures only show the effects that have the largest variations between the subjects. The subjects at clusters 1 and 2 react with generally different insulin concentration sensitivity. One of the specific differences in how monounsaturated fatty acids (MUFA) contributes to insulin concentrations. The cluster 3 with a small number of subjects shows also different reactions to fatty acids and fibers. The figure is plotted with ggplot2 package for R language (v 3.3.2, https://ggplot2.tidyverse.org).

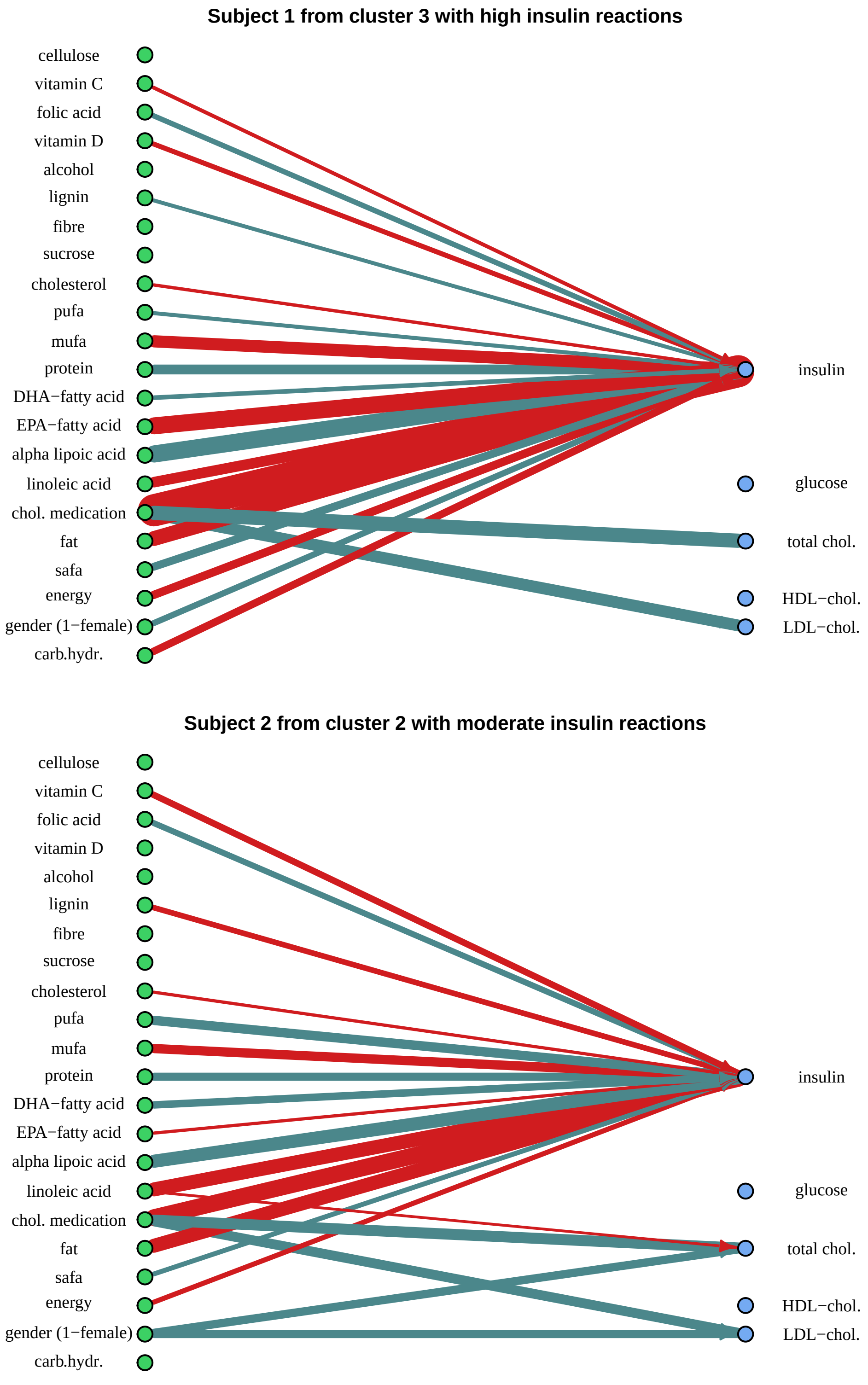

Supplementary Figure S 17: Two subjects were picked from differently reacting clusters and their personally predicted reaction graphs show differences from the general behaviour. The figure is plotted with iGraph package for R language (v 1.2.6, https://igraph.org/r).

Supplementary Table S 8: The most differentiating nutritional effects between the compared subjects.

Subject	Effect	Slope
1	safa -> insulin	-0.72 [-2.5; 1.02]
2	safa -> insulin	-0.44 [-2.15; 1.37]
1	lignin -> insulin	-0.36 [-1.75; 0.75]
2	lignin -> insulin	0.51 [-0.52; 1.71]
1	vitamin C -> insulin	0.34 [-1.1; 1.82]
2	vitamin C -> insulin	0.59 [-0.67; 1.91]
1	vitamin D -> insulin	0.45 [-0.41; 1.43]
2	vitamin D -> insulin	0.16 [-0.72; 1.02]
1	energy -> insulin	0.75 [-2.35; 4.01]
2	energy -> insulin	0.46 [-2.57; 3.62]
1	EPA-fatty acid -> insulin	1.53 [-0.02; 3.29]
2	EPA-fatty acid -> insulin	0.29 [-1.39; 1.88]

Supplementary Table S 9: Average concentration levels during the 12-week study for the compared subjects.

Subject	HDL-chol.	LDL-chol.	Insulin	Total chol.	Glucose
1	1.21	3.29	29.38	5.43	6.85
2	0.90	3.58	22.04	5.12	6.90

References

Lankinen, M., U. Schwab, M. Kolehmainen, J. Paananen, K. Poutanen, H. Mykkanen, T. Seppanen-Laakso, H. Gylling, M. Uusitupa, and M. Oresic. 2011. "Whole grain products, fish and bilberries alter glucose and lipid metabolism in a randomized, controlled trial: the Sysdimet study." *PLoS ONE* 6 (8): e22646.

Limpert, Werner A., Eckhard AND Stahel. 2011. "Problems with Using the Normal Distribution – and Ways to Improve Quality and Efficiency of Data Analysis." *PLOS ONE* 6 (7): 1–8. https://doi.org/10.1371/journal.pone.0021403.

Piironen, Juho, and Aki Vehtari. 2017. "Sparsity Information and Regularization in the Horseshoe and Other Shrinkage Priors." *Electron. J. Statist.* 11 (2): 5018–51. https://doi.org/10.1214/17-EJS1337SI.
